# Vibrational Spectroscopy of Hexahalo Complexes

**DOI:** 10.1021/acs.inorgchem.2c00125

**Published:** 2022-04-05

**Authors:** Stewart F. Parker, Kenneth P. J. Williams, Timothy Smith, Anibal J. Ramirez-Cuesta, Luke L. Daemen

**Affiliations:** † ISIS Facility, STFC Rutherford Appleton Laboratory, Chilton, Didcot OX11 0QX, U.K.; ‡ School of Chemistry, University of Glasgow, Joseph Black Building, Glasgow G12 8QQ, U.K.; § Renishaw plc, New Mills, Gloucestershire, Wotton-under-Edge GL12 8JR, U.K.; ∥ Neutron Science Directorate, Oak Ridge National Laboratory, Oak Ridge, Tennessee 37831, United States

## Abstract

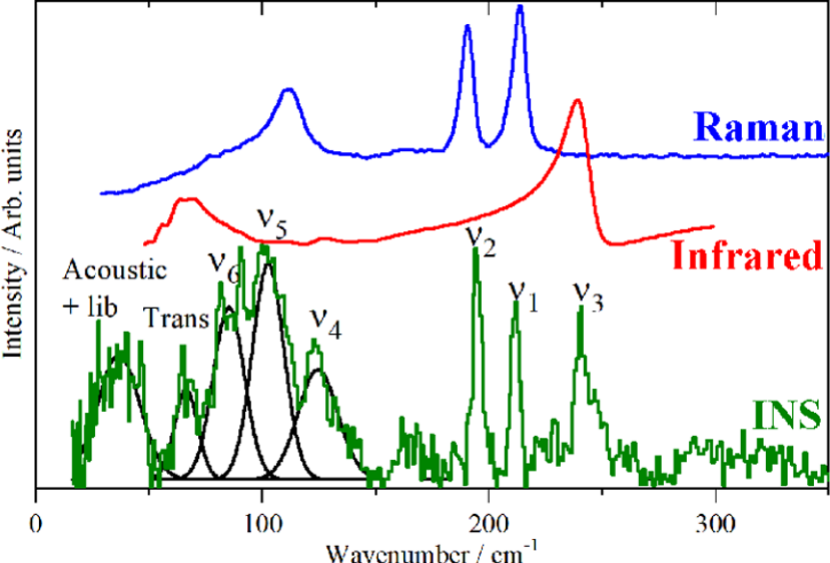

Halogenated inorganic complexes *A*_*x*_[*M*Hal_*y*_] (*A* = alkali metal or alkaline earth, *M* = transition or main group metal, *x* = 1–3, and *y* = 2–9) are an archetypal class of compounds that provide entry points to large areas of inorganic and physical chemistry. All of the hexahalo complexes adopt an octahedral, *O*_h_, symmetry (or nearly so). Consequently, one of the bending modes is forbidden in both the infrared and Raman spectra. In the solid state, many of the complexes crystallize in the cubic space group *Fm*3̅*m*, which preserves the octahedral symmetry. Even for those that are not cubic, the octahedral symmetry of the [*M*Hal_6_]^*n*−^ ion is largely retained and, to a good approximation, so are the selection rules. In the present work, we show that by using the additional information provided by neutron vibrational spectroscopy, in combination with conventional optical spectroscopies, we can generate complete and unambiguous assignments for all the modes. Comparison of the experimental and calculated transition energies for the systems where periodic-density functional theory was possible (i.e., those for which the crystal structure is known) shows that the agreement is almost quantitative. We also provide a linear relationship that enables the prediction of the forbidden mode.

## Abstract 2

Halogenated inorganic complexes provide entry to large areas of inorganic chemistry. All of the hexahalo complexes adopt an octahedral symmetry, so one of the bending modes is forbidden in both the infrared and Raman spectra. By using neutron vibrational spectroscopy, we can generate unambiguous assignments for all the modes. Comparison of experimental and calculated transition energies shows that the agreement is almost quantitative. We also provide a linear relationship that enables prediction of the forbidden mode.

## Introduction

Halogenated inorganic complexes *A*_*x*_[*M*Hal_*y*_] (*A* = alkali metal or alkaline earth, *M* = transition or main group metal, *x* = 1–3, and *y* = 2–9) are an archetypal class of compounds that provide entry points to large areas of inorganic and physical chemistry.^1,2^ Of particular current interest for photovoltaic applications is CsPbBr_3_^3^ and the closely related hybrid organic–inorganic perovskites that are derivatives of CsPb*X*_3_ (*X* = Cl, Br, or I).^4−6^ For light-emitting applications, Cs_4_PbBr_6_^7^ has emerged as a very promising candidate.^8^ In other fields, K_2_[PtCl_6_] is used as a catalyst precursor,^9^ and cryolite, Na_3_[AlF_6_], as a molten salt is used as a solvent for aluminum oxides in the electrolytic refining of aluminum from its ores.^10^

As might be expected, the vibrational spectra of these materials have been extensively investigated for many years: the definitive reference book by Nakamoto^11^ lists the transition energies of almost 150 such compounds for the hexahalo complexes alone. However, for only two compounds, K_2_[PtCl_6_] and K_2_[IrCl_6_], have all of the fundamental modes of the hexahalo ions been observed and assigned.^12^ The reasons for this are both pragmatic and fundamental: as the halogen becomes heavier, the modes shift down in energy and become increasingly difficult to observe with conventional infrared and Raman spectroscopies. All of the hexahalo complexes adopt an octahedral, *O*_h_, symmetry (or nearly so). Consequently, one of the bending modes is forbidden in both the infrared and Raman spectra. In the solid state, many of the complexes crystallize in the cubic space group *Fm*3̅*m* (no. 225), which preserves the octahedral symmetry. Even for those that are not cubic, the octahedral symmetry of the [*M*Hal_6_]^*n*−^ ion is largely retained and, to a good approximation, so are the selection rules.

In the present work, we show that by using the additional information provided by neutron vibrational spectroscopy^13^ [inelastic neutron scattering, (INS)], in combination with conventional optical spectroscopies, we can generate complete and unambiguous assignments for all the modes. Where the structure is available, we support the assignments with density functional theory calculations of the complete primitive cell.

### INS Spectroscopy

**Figure 1:**
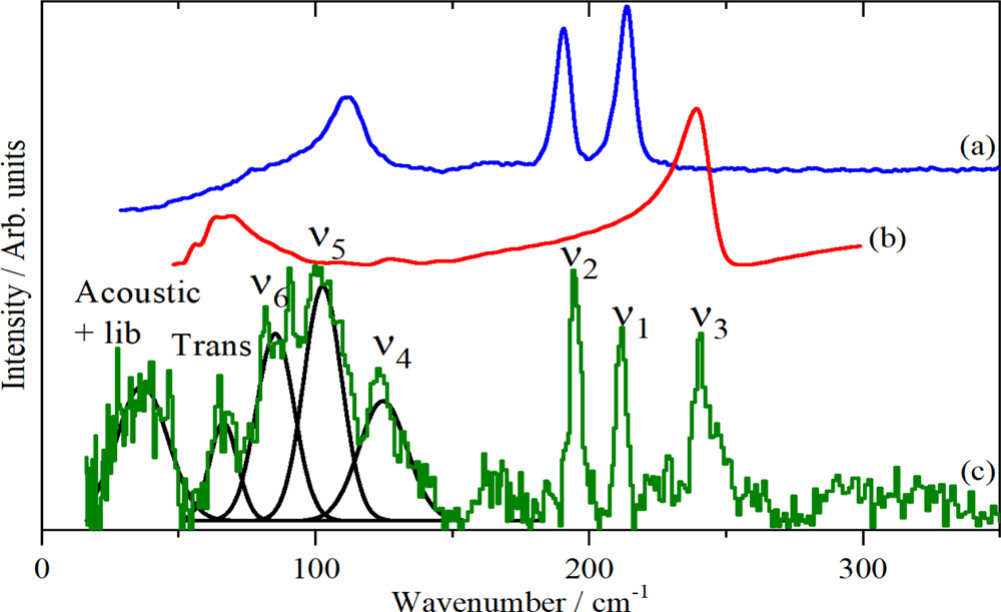
Vibrational spectra of K_2_[PtBr_6_]: (a) Raman recorded with 785 nm excitation at room temperature, (b) infrared at room temperature, and (c) INS recorded on TOSCA at <20 K.

INS spectroscopy^13^ is a form of vibrational spectroscopy, where the inelastic scattering event occurs between the incident neutron and an atomic nucleus. This has a number of consequences, and for the present purposes the key one is that there are no selection rules and all modes are allowed. This is illustrated in Figure 1 for K_2_[PtBr_6_], which crystallizes^14^ in the cubic face centered space group *Fm*3̅*m* with the complex ion on a site of *O*_h_ symmetry. Table 1 lists the modes, the spectral activity, their numbering, and the symmetry for an *A*_2_[*M*Hal_6_] material with an *Fm*3̅*m* symmetry and one formula unit in the primitive cell. From Table 1, it can be seen that there should be no coincidences in the infrared and Raman spectra, and this is indeed the case in Figure 1. In contrast, all the modes are allowed in the INS spectrum and all are seen, including those forbidden in the infrared and Raman spectra. This highlights the complementarity of the three forms of spectroscopy: symmetry assignments are unambiguous from the infrared and Raman spectra and, by inspection, the optically forbidden modes may be located from the INS spectrum.

**Table 1:** Modes, Spectral Activity, Numbering, and Symmetry for an *A*_2_[*M*Hal_6_] Material with a *Fm*3̅*m* Symmetry, One Formula Unit in the Primitive Cell, the Complex Ion on the *O*_h_ Site, and the Counterion on the *T*_d_ Site

			activity		
mode	description	symmetry	IR	R	INS
ν_1_	symmetric M–Hal stretch	*A*_1g_	×	√	√
ν_2_	symmetric M–Hal stretch	*E*_g_	×	√	√
ν_3_	asymmetric M–Hal stretch	*T*_1u_	√	×	√
ν_4_	asymmetric Hal–M–Hal bend	*T*_1u_	√	×	√
ν_5_	symmetric Hal–M–Hal bend	*T*_2g_	×	√	√
ν_6_	asymmetric Hal–M–Hal bend	*T*_2u_	×	×	√
ν_7_	libration	*T*_1g_	×	×	√
ν_8_	optic translation	*T*_1u_	√	×	√
ν_9_	optic translation	*T*_2g_	×	√	√
ν_10_	acoustic translation	*T*_1u_	×	×	√

The intensity, *S*(*Q*,ω), is determined by the total scattering cross section of the atom and the amplitude of motion of the atoms in the vibrational mode. The cross section is both element and isotope dependent, for the halogens^15^ it is: ^19^F = 4.0, ^35^Cl = 21.8, ^37^Cl = 1.2, ^79^Br = 5.96, ^81^Br = 5.84, and ^127^I = 3.8 b [15] (1 b = 10^–28^ m^2^). For comparison, ^1^H = 82.0 b and this in combination with it being the lightest element (and hence having the largest amplitude of motion) is the reason that INS spectroscopy of chemical systems is dominated by studies of hydrogenous materials.^13^

However, it can be seen that the majority Cl isotope, ^35^Cl (75.8%), has an appreciable total scattering cross section of 21.8 b, which enabled the INS spectra of the K_2_[PtCl_6_] and K_2_[IrCl_6_]^12^ complexes to be recorded in a reasonable time (12 h or so). The other halogens are much less favorable. The smaller mass of F relative to Cl means that the amplitude of motion (and hence INS intensity) is larger but, until recently, this was insufficient to compensate for the much smaller cross section even with a 20 g sample and a measurement time of 24 h, see Figure S1.

As Figure 1 shows, this situation has changed in recent years. TOSCA at ISIS has undergone an upgrade that has greatly boosted the incident flux in the region of interest below 1000 cm^–1^.^16^ Even more significantly, a new instrument, VISION^17^ at the spallation neutron source (SNS, Oak Ridge, TN, USA), has become operational, which has unprecedented sensitivity. As we will show, these instruments now allow even the most unfavorable systems, those containing Br or I, to be routinely measured.

## Experimental Section

### Materials

K_2_[SiF_6_], Na_2_[SiF_6_], K_2_[TiF_6_], K[PF_6_], and Na_3_[AlF_6_] were purchased from Aldrich. K_2_[ReCl_6_], K_2_[IrCl_6_], K_2_[PtBr_6_], K_2_[PtI_6_] were purchased from Alfa Aesar. All compounds were used as received. It should be noted that in addition to the materials listed, several other compounds were purchased from reputable vendors; however, their spectra were incompatible with the stated composition. *Caveat emptor*!

### Infrared Spectroscopy

Room-temperature infrared spectra (50–4000 cm^–1^, 4 cm^–1^ resolution, 64 scans, 8 × zero filling) were recorded with a Bruker Vertex 70 Fourier transform infrared spectrometer using a Bruker Platinum single reflection attenuated total internal reflection accessory. Some spectra were also measured in transmission as polyethylene discs (∼1 wt % in low density polyethylene). The low wavenumber limit (∼50 cm^–1^) is determined by the very low output of the globar infrared source at these energies, resulting in almost no signal.

### Raman Spectroscopy

Raman spectra were measured with a variety of instruments and excitation wavelengths. FT-Raman spectra were recorded at room temperature with a Bruker MultiRam spectrometer using 1064 nm excitation (500 mW laser power and 1024 scans at 1 or 4 cm^–1^ resolution). Multi-wavelength (405, 532, 633, and 785 nm) Raman spectra were recorded with a Renishaw InVia Raman microscope system. Some spectra were also obtained with a Bruker Senterra system using 532 and 785 nm excitation. Variable temperature (13–300 K) Raman spectra were recorded with a modified Renishaw InVia spectrometer using 785 nm excitation that has been previously described.^18^ The low wavenumber limit (∼40 cm^–1^ for all the spectrometers) is set by the cutoff of the notch filter used to reject the laser line.

### INS Spectroscopy

INS spectra were recorded with the broad band, high-resolution indirect geometry spectrometers TOSCA^16^ (and its predecessor TFXA^19^) at ISIS and VISION^17^ at SNS. At ISIS, the sample size was typically ∼10 g and measurement times ranged from 6 to 24 h. VISION is considerably more sensitive, so sample sizes were ∼5 g with a few hours of measurement time. All spectra were measured below 20 K unless noted otherwise.

### Computational Studies

Dispersion-corrected periodic density functional theory (DFT-D) calculations were carried out with CASTEP (version 17.21).^20^ On-the-fly generated norm-conserving pseudopotentials were used with the PBE^21^ functional with the Tkatchenko–Scheffler (TS) dispersion correction scheme^22^ using the generalized gradient approximation (GGA). The plane wave cutoffs and the Brillouin zone sampling of electronic states are given in Table S12. The equilibrium structure was obtained by BFGS geometry optimization and converged to, typically, |0.001| eV Å^–1^. Phonon frequencies were obtained by diagonalization of the dynamical matrix, computed using density functional perturbation theory,^23^ to compute the dielectric response and the Born effective charges and, from these, the mode oscillator strength tensor and infrared absorptivity were calculated. Raman intensities were calculated by a finite displacement method.^24^ In addition to the calculation of transition energies at zero wavevector, phonon dispersion was also calculated along high symmetry directions throughout the Brillouin zone. For this purpose, dynamical matrices were computed on a regular grid of wavevectors throughout the Brillouin zone, and Fourier interpolation was used to extend the computed grid to the desired fine set of points along the high-symmetry paths.^25^ The atomic displacements in each mode, that are part of the CASTEP output, enable visualization of the modes in Materials Studio^26^ to aid assignments and are also all that is required to generate the INS spectrum via ACLIMAX (version 6.0.0 LE).^27^ It is emphasized that the calculated transition energies have not been scaled.

## Results

In this section, we will provide examples of the spectra of hexahalo complexes for all of the halogens. The assignments will be supported, where possible, by DFT calculations of the primitive cell. We will conclude by comparing all of the experimental and DFT transition energies, in order to provide estimates of the reliability of the DFT results for each mode. The observed transition energies and assignments are given in Table 2. The calculated transition energies at the Brillouin zone Γ-point and the dispersion curves are provided as tables and figures in the Supporting Information.

**Table 2:** Observed and Calculated Transition Energies (cm^–1^) of the *A*_*x*_[*M*Hal_*y*_] Complexes

	K_2_[SiF_6_]		Na_2_[SiF_6_]		K_2_[TiF_6_]		K[PF_6_]		Na_3_[AlF_6_]		K_2_[ReCl_6_]	K_2_[IrCl_6_]	K_2_[PtCl_6_]		K_2_[PtBr_6_]	K_2_[PtI_6_]	
	exp	cal	exp	cal^b^	exp	cal	exp	cal^b^	exp	cal	exp	exp	exp	cal	exp	exp	cal^b^
SG	*Fm*3̅*m*		*P*321		*P3̅m1*		*C*2/*n*		*P*2_1_/*n*		*Fm*3̅*m*	*Fm*3̅*m*	*Fm*3̅*m*		*Fm*3̅*m*	*P*2_1_/*c*	
site	*O*_h_		*C*_3_, *D*_3_		*D*_3d_		*C*_i_				*O*_h_	*O*_h_	*O*_h_				
ν_1_	656	632	663	645	614	592	751	711	553	541	359	350	346	315	223	151	146
ν_2_	478	471	479	469	450	452	588	539	396	415	302	310	325	295	206	132	127
							579										
							573										
ν_3_	722	718	707	729	532	552	786	789	560	590	315	330	346	318	251	186	177
						542											
ν_4_	477	460	492	481	308	298	545	557	414	395	166	177	183	188	135	95	89
			473		274	294			394								
ν_5_	408	392	408	396	305	286	485	476	344	327	174	166	164	167	112	77	71
					292	260	479										
							474										
ν_6_	272	258	284	282	198	179	324,	313	278	272	132	143	147	140	96	62	57
					187		318,		257								
		,			178		310										
ν_7_ lib	81	68			81	86						48	55	28		31	32
						55											
ν_8_ IR trans	114	128				115					75/106^a^		83	80		111	87
						93											
ν_9_ Raman trans	125	124				124					80		80	82			
						97											

a LOTO splitting.

b Average of site and factor group components.

### Fluoro Complexes

We will begin with K_2_[SiF_6_] because this is the simplest material as it has a cubic (*Fm*3̅*m*) symmetry;^28^ systems with a lower symmetry (Na_2_[SiF_6_], K_2_[TiF_6_], K[PF_6_], and Na_3_[AlF_6_]) will then be considered.

#### K_2_[SiF_6_]

**Figure 2:**
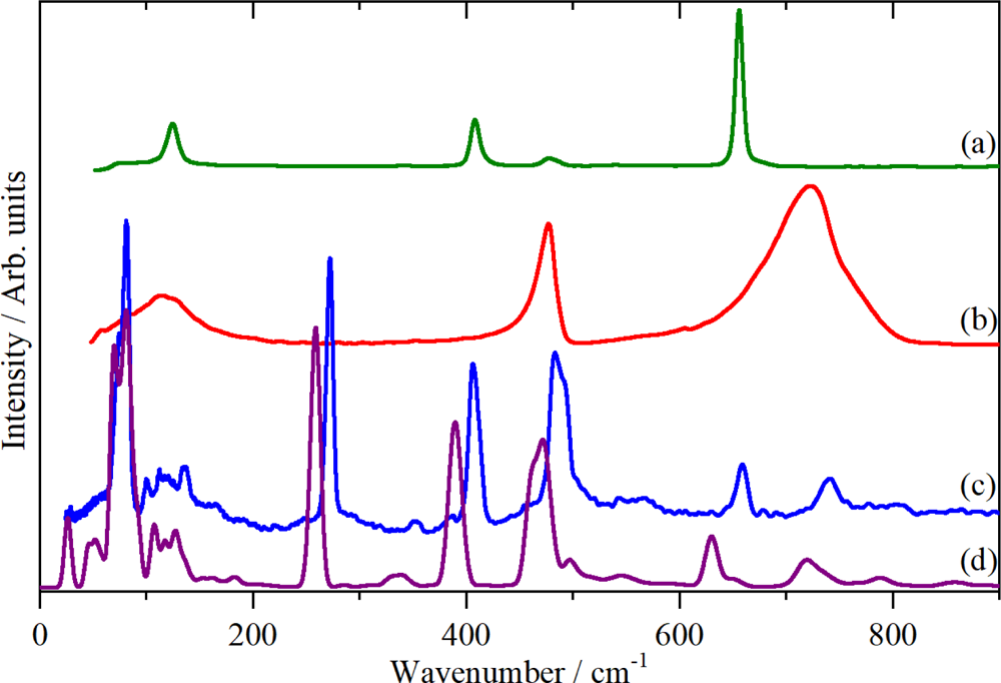
Vibrational spectra of K_2_[SiF_6_]: (a) Raman recorded with 1064 nm excitation at room temperature, (b) infrared at room temperature, and (c) INS recorded on TOSCA at <20 K and (d) INS spectrum generated from the CASTEP output in space group *Fm*3̅*m*.

Figure 2 shows the Raman, infrared, and INS spectra of the material. The combination of the three techniques with the cubic symmetry means that the assignment is straightforward, as listed in Table 2. The only noteworthy feature is that ν_2_ and ν_4_ are almost accidentally degenerate. The assignments are confirmed by comparison with the INS spectrum generated from the DFT calculation (Table S1 and Figure S2), Figure 2d.

#### Na_2_[SiF_6_]

In contrast to the potassium salt, the structure of the sodium salt is still debated with triclinic, *P*1,^29^ and trigonal, *P*321,^30,31^ structures having been proposed. Both structures have *Z* = 3, (*Z* is the number of formula units in the unit cell), but the site symmetry is different: *C*_1_ in *P*1 and *C*_3_ and *D*_3_ in *P*321. In *C*_1_, all degeneracies are formally removed and all modes are infrared and Raman allowed, in *C*_3_ and *D*_3_, *T* modes split to *A* + *E* and all modes are allowed. However, as usual, both proposed structures have [SiF_6_]^2–^ ions with an almost *O*_h_ symmetry, so the optical selection rules would still be expected to be largely valid. The correlation table is given in Table S2.

**Figure 3:**
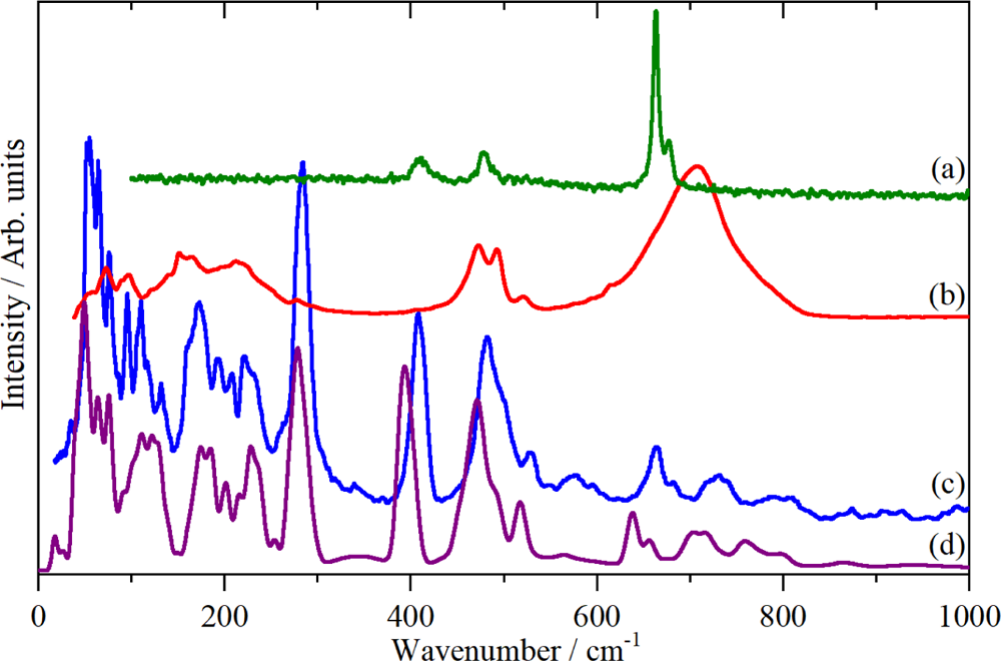
Vibrational spectra of Na_2_[SiF_6_]: (a) Raman recorded with 1064 nm excitation at room temperature, (b) infrared at room temperature, (c) INS recorded on VISION at <20 K, and (d) INS spectrum of the *P*321 structure generated from the CASTEP output.

Figure 3a–c shows the Raman, infrared, and INS spectra and Figure 3d shows the results of a DFT calculation based on the *P*321^20^ structure. The Raman spectrum is very similar to that of K_2_[SiF_6_] and is assigned similarly: ν_1_, ν_2_, and ν_5_ (from high to low). The infrared and INS spectra are more interesting and the lower symmetry is particularly evident in the lattice mode region below 250 cm^–1^ where there are many more modes, reflecting that there are now nine, rather than three, ions in the primitive cell. ν_3_ and ν_4_ are clearly seen in the infrared spectrum, as with K_2_[SiF_6_], ν_2_ and ν_4_ are almost coincident, the latter is split by the lower site symmetry. There is no evidence for ν_1_ or ν_5_ in the infrared spectrum but there is a very weak band at 279 cm^–1^ which the INS shows is ν_6_. The calculated (Table S3 and Figure S3), INS spectrum, Figure 3d, is in reasonable agreement with the experimental one and thus supports (but does not prove) the *P*321^30,31^ structure.

The mode animations from the calculations show that the low site symmetry, in particular the absence of a center of symmetry, results in a partial breakdown of the *O*_h_ selection rules. This is apparent from the correlation table for the complex assuming the *P*321 structure, as listed in Table S3. This is also shown by one of the components of ν_2_ having significant infrared activity and the mixing of the translational and librational modes, particularly for the lower symmetry *C*_3_ site.

A series of 11 hexafluorosilicate salts were previously measured.^32^ The assignments are largely in agreement with those given here, with the exception of ν_6_. This was estimated at 325 cm^–1^, whereas the present work shows it to be at 279 and 272 cm^–1^ in the Na and K salts, respectively.

#### K_2_[TiF_6_]

**Figure 4:**
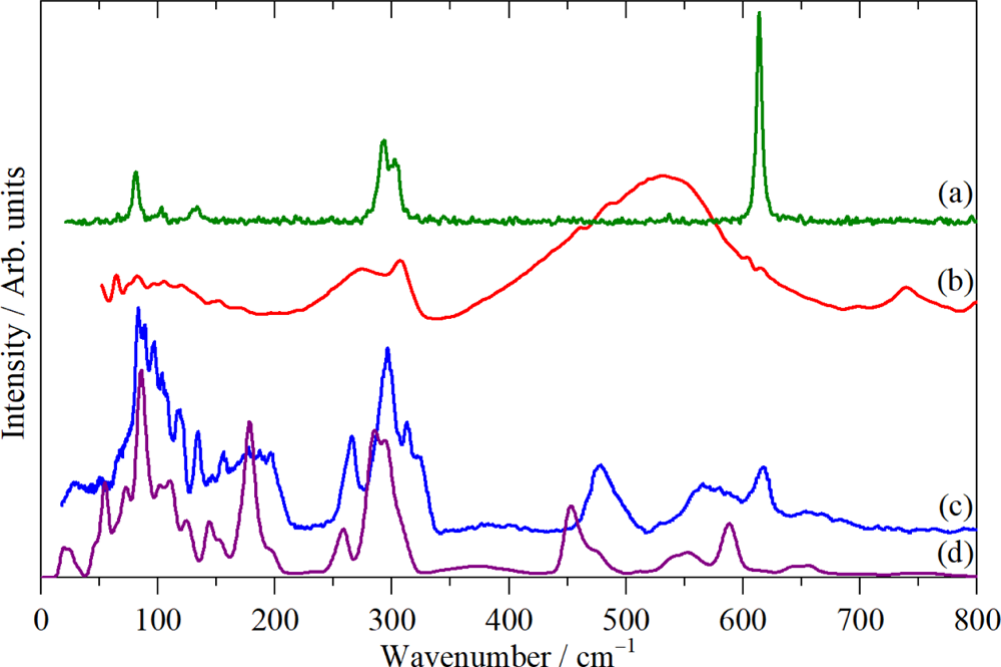
Vibrational spectra of K_2_[TiF_6_]: (a) Raman recorded with 1064 nm excitation at room temperature, (b) infrared at room temperature, (c) INS recorded on VISION at <20 K, and (d) INS spectrum generated from the CASTEP output in space group *P*3̅*m*1.

At room temperature, this material crystallizes in the trigonal space group *P*3̅*m*1 (no. 164) with *Z* = 1 and the complex ion on a *D*_3d_ site.^33^ The infrared and Raman spectroscopy have been comprehensively investigated^34^ and all the modes observed except for ν_2_ and ν_6_. Figure 4 shows the observed spectra and the calculated INS spectra (Table S4 and Figure S4).

From the INS spectrum, by inspection ν_1_ and ν_3_ are seen at 618 and 550 cm^–1^, ν_2_ is apparent at 477 cm^–1^ and ν_6_ at 150–200 cm^–1^. ν_4_ and ν_5_ are almost coincident and account for the complex feature at 250–350 cm^–1^. The calculated spectrum is in reasonable agreement with the experimental data, but the agreement is not as good as would be expected. The structure^33^ was determined at room temperature, and it is possible that there is a phase transition at lower temperatures. Nonetheless, the result is sufficiently close to enable unambiguous assignments to be made. The asymmetric shape of ν_2_ and the feature at 650 cm^–1^ are the result of significant dispersion in ν_2_ and ν_3_, respectively, see Figure S4.

#### K[PF_6_]

**Figure 5:**
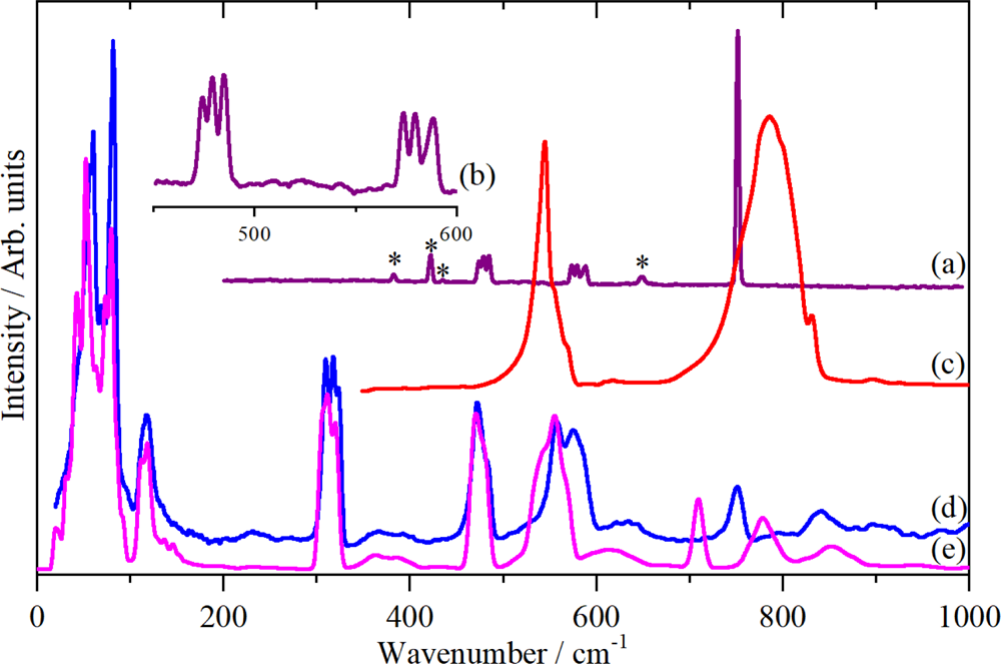
Vibrational spectra of K[PF_6_] in phase III: (a) Raman recorded with 785 nm excitation at 45 K, (b) ordinate (×12) and abscissa (×2) expanded Raman spectrum at 45 K in the ν_2_ and ν_4_ region, (c) infrared at 150 K, (d) INS recorded on VISION at <20 K, and (e) INS spectrum generated from the CASTEP output of the primitive cell of the *C*2/*n* space group. [The asterisks in (a) denote bands from the sapphire window].

This material has an interesting phase diagram.^35−38^ The room-temperature phase I is cubic (*Fm*3̅*m*) but with tilted, orientationally disordered PF_6_ octahedra. At around 256 K (the values vary in the literature), the monoclinic (*A*2/*n*) phase II forms. Below 210 K, the low-temperature phase III occurs, which is also monoclinic, but *C*2/*n* with *Z* = 4. The atomic coordinates are only available in a thesis^36^ and are reproduced in Table S5. Figure 5 shows the Raman, infrared, and INS spectra of K[PF_6_] in phase III. The infrared and Raman spectra are in good agreement with previous works,^38^ but because the structure was not known and in the absence of the INS spectrum at that time, only tentative assignments were possible.

From the structure,^36^ the complex ion is known to be on a *C*_i_ site. Despite the low-site symmetry, the P–F bonds are all almost equal (1.602, 1.603, and 1.608 Å), consequently the *O*_h_ selection rules are largely obeyed, making the assignment straightforward: ν_1_ at 544 cm^–1^, ν_2_ at 588/578/574 cm^–1^, ν_3_ at 786 cm^–1^, ν_4_ at 509 cm^–1^, ν_5_ at 485/479/473 cm^–1^, and ν_6_ at 324/318/310 cm^–1^. The calculated INS spectrum is in excellent agreement apart from the P–F stretch modes, which are slightly underestimated (Table S6 and Figure S5). This may be a result of the calculated P–F distances being slightly longer (1.606, 1.607, and 1.609 Å) than the experimental values.

#### Na_3_[AlF_6_]

This material is better known as the mineral cryolite and the spectra (including the INS) have been comprehensively assigned previously.^39^ The spectra are shown in Figure S6.

### Chloro Complexes

**Figure 6:**
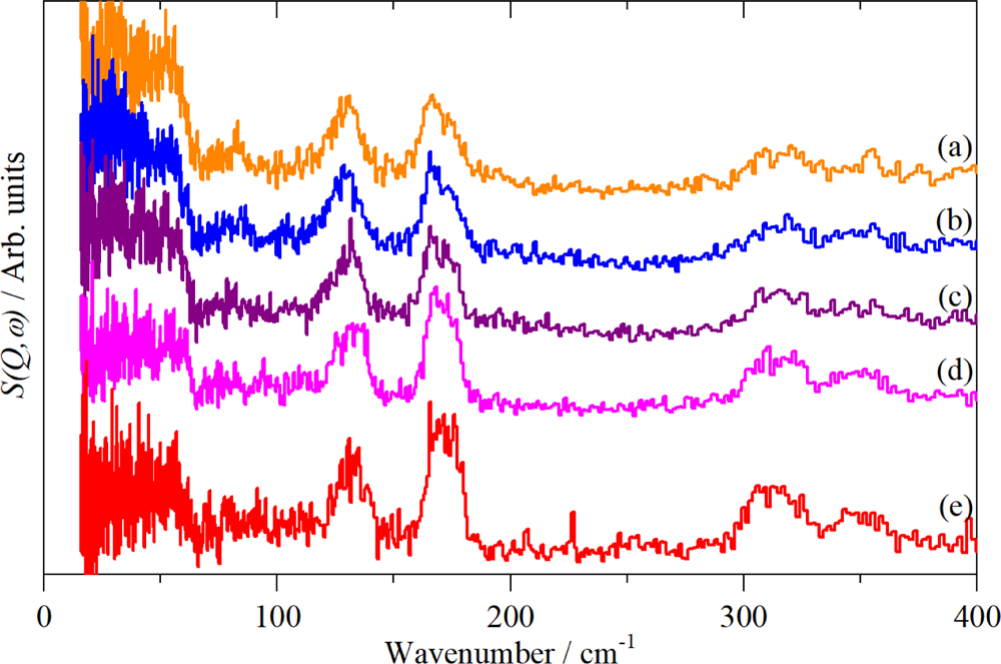
Variable temperature INS spectra of K_2_[ReCl_6_] recorded on TFXA: (a) 120 K, cubic, (b) 107 K, tetragonal, (c) 90 K, monoclinic, (d) 25 K, possibly tetragonal, and (e) 5 K, unknown structure but antiferromagnetically ordered.

**Figure 7:**
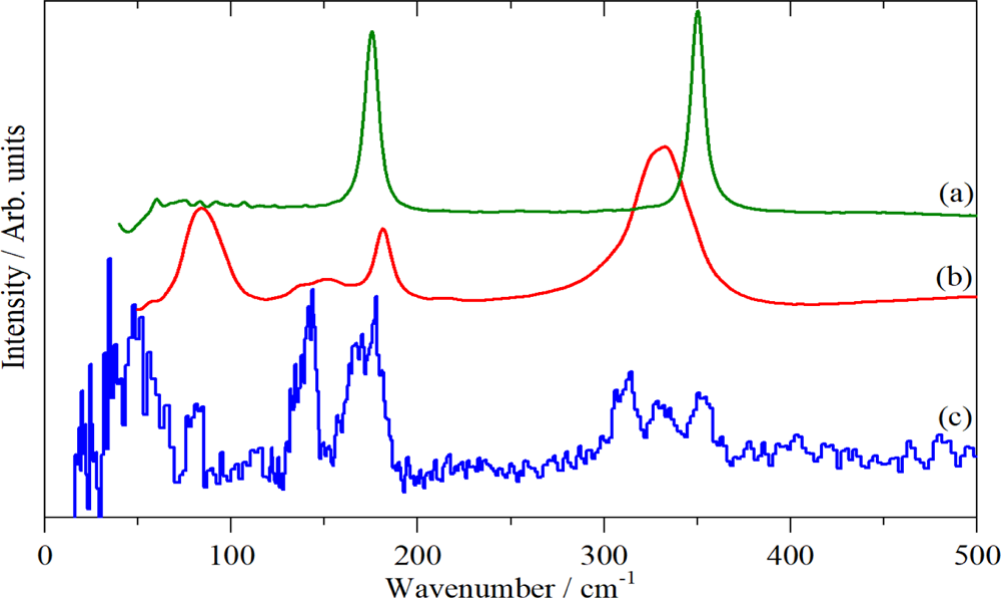
Vibrational spectra of K_2_[IrCl_6_]: (a) Raman (785 nm) at room temperature, (b) infrared as a polyethylene disc at room temperature, and (c) INS at 5 K recorded on TFXA.

As discussed in the Introduction, the chloro complexes are the most favorable for INS studies because of the large incoherent cross section of ^35^Cl. Despite this, inspection of the INS spectra of the chloro complexes (Figures 6, 7 and S7) shows that the signal-to-noise ratio is markedly inferior to that of the fluoro complexes (Figures 2–5 and S6). The chloro spectra were measured with a first generation instrument (TFXA^13^). The spectra of the fluoro complexes emphasizes the enormous gains in flux and sensitivity that state-of-the-art instrumentation (the upgraded TOSCA^16^ and VISION^17^) have achieved.

The INS spectra of K_2_[PtCl_6_] and K_2_[IrCl_6_] have been previously reported;^12^ however, the analysis only considered the isolated [*M*Cl_6_]^2–^ ion with an empirical force field. We have re-analyzed the spectra with DFT and present new data for K_2_[ReCl_6_].

#### K_2_[ReCl_6_]

While K_2_[ReCl_6_] is cubic at room temperature,^40^ it undergoes a series of phase transitions as the temperature is lowered:^41,42^
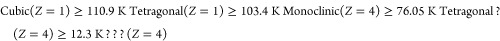


Below 12.3 K, the compound orders antiferromagnetically with an associated structural change,^43^ neither of the low-temperature structures is known. The spectroscopy has been the subject of an extraordinarily detailed investigation by O’Leary and Wheeler^42^ that has characterized the spectra in each of the phases. The work has shown that the [ReCl_6_]^2–^ ion remains octahedral in all the phases and that the phase transitions occur as a result of successive small tilts of the octahedra.^42−44^

The spectra in the cubic phase, Figure S9, are in complete agreement with the literature.^42,45^ The INS spectra in each of the five phases are shown in Figure 6. Apart from minor differences in intensity as a result of the changing Debye–Waller factor, the spectra are essentially identical. This is consistent with the various phases differing only in the tilts of the ReCl_6_ octahedra. The absence of selection rules means that all modes are allowed in all phases. As the overall structure does not change by very much, the INS spectra are largely invariant to the phase.

We were unable to calculate the spectra for K_2_[ReCl_6_] as the structure of the low temperature phase is unknown. Attempts to model the spectra using the cubic room temperature structure were unsuccessful. The dispersion curves showed negative values at the Γ and the X-points in the Brillouin zone for the librational modes, that is, they are imaginary modes, see Figure S10. This is in complete agreement with the work of O’Leary and Wheeler^42^ who concluded that the phase transitions are driven by the softening of these modes.

#### K_2_[IrCl_6_]

K_2_[IrCl_6_] is reported to be cubic down to the Néel temperature of 3 K, where it magnetically orders.^46^ The spectroscopy of K_2_[IrCl_6_] has been investigated^45,47,48^ and our infrared and Raman spectra, Figure 7a,b, are in general agreement with the literature. The infrared and Raman spectra show that ν_1_ occurs at 350 cm^–1^, ν_3_ at 330 cm^–1^, and ν_2_ at 310 cm^–1^. ν_2_ has only been previously observed in solution by Raman spectroscopy.^37^ The INS spectrum, Figure 7c, confirms that ν_4_ and ν_5_ are almost accidentally degenerate and ν_6_ is clearly seen at 143 cm^–1^. It is surprising that the infrared spectrum shows two bands at 135 and 155 cm^–1^, both are close to the position of ν_6_ but for it to be activated would require that the cubic symmetry is broken and there is no other evidence for this. The *T*_1u_ translational mode is the intense infrared band at 85 cm^–1^ (also seen in the INS) and we assign the 135 cm^–1^ band as a combination of this mode and the *T*_1g_ translational mode at 50 cm^–1^. The product (*T*_1u_ ⊗ *T*_1g_ = *A*_1u_ + *E*_u_ + *T*_1u_ + *T*_2u_) has a *T*_1u_ component that is allowed in the infrared spectrum. The 155 cm^–1^ band is more difficult, we note that room temperature corresponds to ∼200 cm^–1^, thus there will be appreciable population of the modes, and hence we assign this mode to a hot band (ν_3_–ν_5_): 155 = 330–175 cm^–1^. As with the 135 cm^–1^ band, the product (*T*_1u_ ⊗ *T*_2g_ = *A*_2u_ + *E*_u_ + *T*_1u_ + *T*_2u_) has a *T*_1u_ component that is infrared allowed.

#### K_2_[PtCl_6_]

The spectra of K_2_[PtCl_6_] are shown in Figure S7 and the assignments are given in Table 2. The calculated data are in Table S7 and Figure S8.

### Bromo and Iodo Complexes

The combination of small cross sections with large mass means that the bromo and iodo complexes are the most challenging of the hexahalo quartet for INS spectroscopy. As the modes occur below ∼300 cm^–1^, this has also made observing the modes by infrared spectroscopy non-routine. This arises because infrared spectrometers commonly use KBr optics, which sets the low wavenumber limit to 400 cm^–1^.

#### K_2_[PtBr_6_]

The assignment of the spectra is given in Figure 1 and for ν_1_, ν_2_, ν_3_, and ν_5_ that is unambiguous. Previous work^45,48,49^ assigned ν_4_ at 78 or 128 cm^–1^, and it is apparent that the latter is correct and that the 78 cm^–1^ feature is the infrared active translational mode, ν_8_. The ambiguity arose because ν_4_ is unusually weak in the infrared spectrum.

As with K_2_[ReCl_6_], K_2_[PtBr_6_] also undergoes a series of phase transitions as the temperature is lowered:^50^
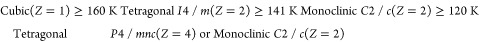


**Figure 8:**
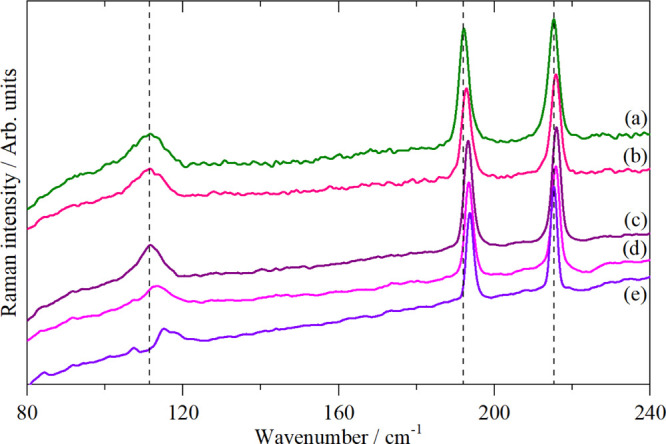
Variable temperature Raman spectra (785 nm excitation) of K_2_[PtBr_6_] at: (a) 250 K, cubic, (b) 220 K, cubic, (c) 156 K, tetragonal, (d) 122 K, monoclinic, and (e) 22 K, tetragonal or monoclinic. The dashed vertical lines indicate the position of ν_1_, ν_2_, and ν_5_ in the cubic phase.

Variable temperature Raman spectra, Figure 8, show minor changes in the position of ν_2_ and almost no change in ν_1_. In the low-temperature phase, ν_5_ has apparently split into three bands, which would favor the *C*2/*c* choice of the space group. This is because with a monoclinic symmetry the threefold degeneracy of the *O*_h_*T* modes is completely removed whereas with a tetragonal symmetry, the *T* modes split to *A* and *E* modes. Unfortunately, this is not conclusive because the presence of more than one molecule in the primitive cell means that a factor group splitting is present and this may account for the additional modes. However, this would also be the case for ν_1_ and ν_2_ and neither shows additional modes, suggesting that the factor group splitting is small. On balance, the spectroscopy favors the monoclinic choice.

An attempt to investigate anharmonicity in K_2_[PtBr_6_] by the observation of an overtone progression, as seen in the ammonium salt,^51^ was unsuccessful (see the Supporting Information).

#### K_2_[PtI_6_]

**Figure 9:**
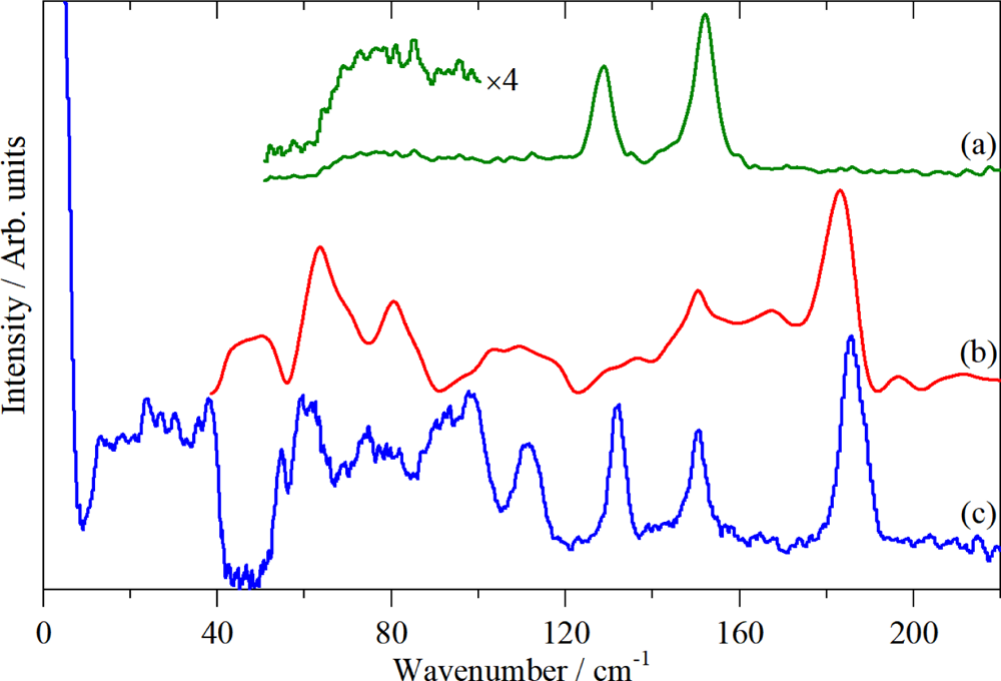
Vibrational spectra of K_2_[PtI_6_]: (a) Raman at room temperature (1064 nm excitation), (b) infrared at room temperature, and (c) INS at 5 K recorded on VISION.

The vibrational spectra are shown in Figure 9. The Raman spectrum is straightforward with ν_1_ and ν_2_ at 152 and 128 cm^–1^, respectively, as seen previously^51,52^ and confirmed by solution polarization measurements.^51^ The intense infrared mode at 184 cm^–1^ is clearly ν_3_.^45,53^ The assignment of the remaining modes is much less obvious. There are two reasons for this: first, the large mass of iodine means that bending modes overlap with the external modes, making the INS spectrum particularly congested. Second, the infrared spectrum is unexpectedly complex, with many more modes than expected and is highly problematic.

**Figure 10:**
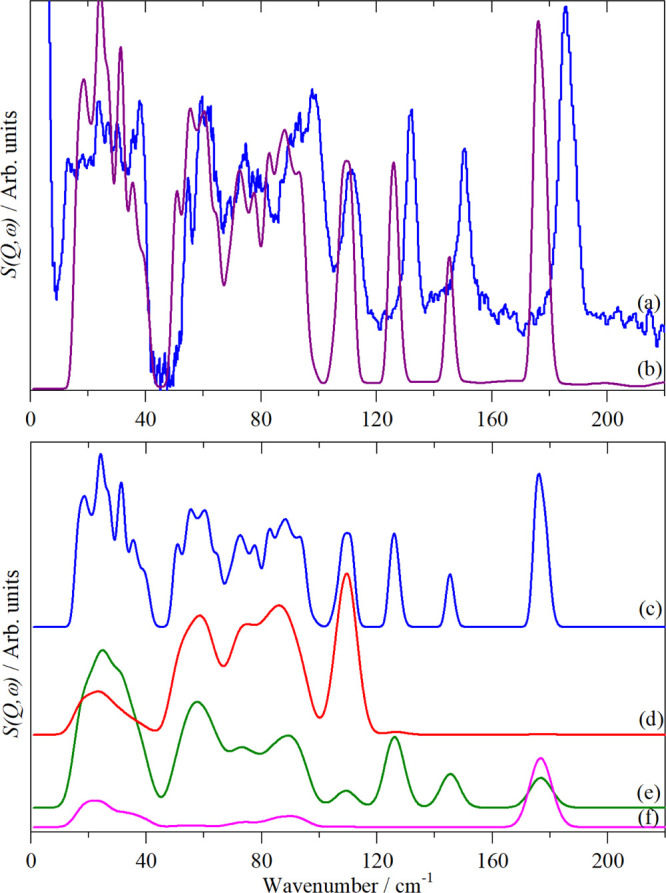
Top panel: (a) INS spectrum of K_2_[PtI_6_] at 5 K recorded on VISION and (b) generated from the CASTEP output of the calculation in the space group *P*2_1_/*c*. Lower panel: contributions (fundamentals only) to the calculated INS spectrum (c) from: (d) K, (e) I, and (f) Pt. (f) is ×2 ordinate expanded relative to (d,e).

This material is unusual in that the room-temperature structure is tetragonal,^54^*Pmnc*, rather than cubic, and *Z* = 2. All attempts to calculate the spectra in *Pmnc* were unsuccessful and resulted in the doubly degenerate librational mode being imaginary. This strongly suggests that below room temperature there is a phase transition to a structure of a lower symmetry. This behavior is reminiscent of K_2_[ReCl_6_] and K_2_[PtBr_6_], where, as the temperature is reduced, a series of tilts of the octahedra result in a monoclinic structure. For K_2_[PtI_6_], tilting the octahedra produces a monoclinic structure with the space group *P*2_1_/*c* (*Z* = 2), as shown in Figure S12. This structure has all real modes across the entire Brillouin zone (see Figure S13 and Table S8), showing that it is dynamically stable. As Figure 10a,b shows, there is good agreement with the experimental spectrum.

The correlation tables for K_2_[PtI_6_] in *Pmnc* and *P*2_1_/*c* are given in Tables S9 and S10. In both space groups, the center-of-symmetry is retained, thus gerade (*g*) and ungerade (*u*) modes in *O*_h_ retain their character in the non-cubic space groups, so the rule of mutual exclusion should still hold. The reduction in symmetry means that ν_6_ is now allowed in the infrared spectrum. More significantly, the K^+^ translational modes now have the same symmetry as the internal modes and so are allowed to mix with all of them.

The INS spectrum can be decomposed into the individual contributions from K, I, and Pt, as shown in Figure 10c–e. These confirm the assignments of ν_1_, ν_2_, and ν_3_: the first two only involve motion of I, while the last also has motion of Pt, exactly as expected for *g* and *u* modes, respectively. These have energies above those of the translational modes, so they do not mix with them and are relatively pure modes.

The Raman spectra^52^ of a series of amine salts of the hexaiodoplatinate ions show a mode around 114 cm^–1^ that was assigned as ν_5_ or a lattice mode. Our Raman spectrum does not show this mode, but there is a strong mode at 111 cm^–1^ in the INS spectrum. Figure 10c–e shows that this mode has a major contribution from K and almost nothing from I, showing that it is a K translational, that is, lattice mode.

The region 40–100 cm^–1^ in the INS spectrum shows three peaks at 60, 75 and 95 cm^–1^, each with some sub-structure. The latter arises from the reduction of symmetry (removal of degeneracies) and the factor group splitting. The elemental decomposition shows that all three modes have substantial contributions from both K and I motions. Mode visualization from the CASTEP calculation shows that these are ν_6_, ν_5_, and ν_4_ (from low to high energy). A close inspection of the Raman spectrum shows a weak band at 78 cm^–1^ consistent with the INS spectrum and it being ν_5_.

The region 0–40 cm^–1^ is not accessible with our infrared or Raman spectrometers (see the Experimental Section) but is readily seen by INS. The mode visualizations and Figure 10d–f show that this region is dominated by the translations and librations.

As stated earlier, the infrared spectrum is problematic. The selection rules predict that the Raman active modes ν_1_, ν_2_, and ν_5_ should not appear in the infrared spectrum. This prediction is not met: ν_1_ and ν_5_ are clearly present, ν_2_ may also be weakly present. After ν_3_, ν_6_ is the strongest mode present, this is unexpected, as although allowed, it would be expected to be weak. Overall, the spectrum suggests a symmetry much lower than *Pmnc*. We currently have no explanation for this result.

## Discussion

One of the aims of this work was to investigate how the combination of INS spectroscopy with infrared and Raman spectroscopies could be used to provide complete assignments for hexahalo systems. This has been comprehensively achieved. For hexafluoro systems, the current generation of INS spectrometers are easily able to measure the spectra. The spectra are spread across the 0–1000 cm^–1^ range, so the modes are relatively well separated which simplifies assignments. The hexachloro systems are also straightforward. The most challenging are the hexabromo and hexaiodo materials, but even these are tractable. The difficulty with these is not acquisition of spectra, but that all of the modes occur in only a 250 cm^–1^ span, meaning that they are highly overlapped. However, even these can largely be assigned by inspection, for example, K_2_[PtBr_6_], Figure 1.

One potential area of concern was the question of how relevant data that was obtained below 20 K, that is, INS was to room-temperature spectra. As Figure 6 shows, the INS spectra are almost invariant with temperature, despite the occurrence of multiple phase transitions. This is a consequence of the absence of selection rules: the phase transitions result in only minor changes to the structure, so the local spatial arrangement of the atoms is largely unchanged, so the spectrum is also unchanged. This means that low temperature INS data can be readily applied to the analysis of room-temperature infrared and Raman spectra, even if there are phase changes below room temperature.

**Figure 11:**
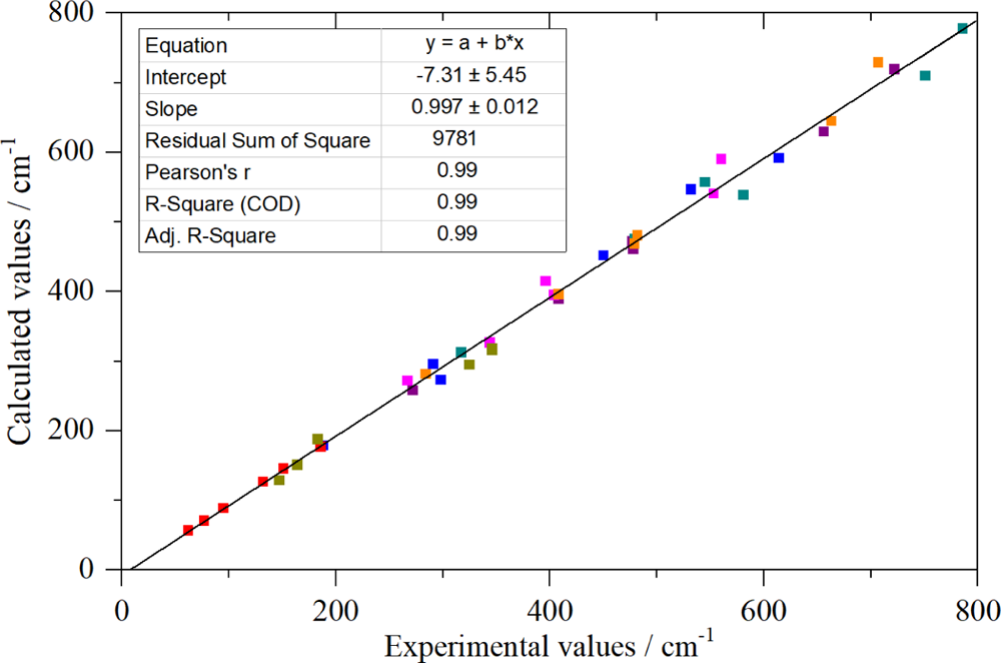
Comparison of experimental and DFT calculated transition energies for hexahalo materials. Key: cyan: K[PF_6_], purple: K_2_[SiF_6_], orange: Na_2_[SiF_6_], blue: K_2_[TiF_6_], magenta: Na_3_[AlF_6_], dark yellow: K_2_[PtCl_6_], and red: K_2_[PtI_6_]. The black line is a least squares fit to the data.

A second aim of the work was to verify that DFT was able to accurately describe these systems. As shown by the comparison of observed and calculated INS spectra in Figures 2c,d, 3c,d, 4c,d, 5d,e, 6c,d, and 10a,b, the description is very good. Figure 11 compares the experimental and calculated transition energies for all of the systems where DFT was possible. It is clear that the agreement is almost quantitative.

**Figure 12:**
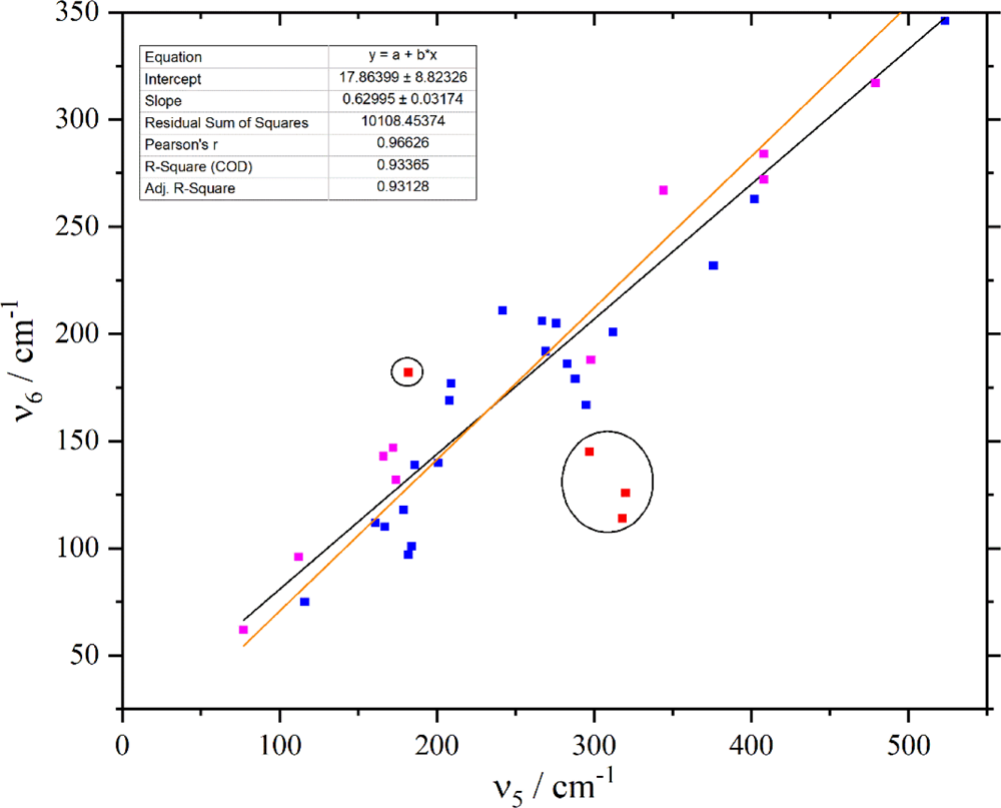
Comparison of ν_5_ and ν_6_ transition energies for hexahalo materials. Points in magenta are from the present work (Table 2) and those in blue are from the literature (see Table S11 for values and references). The black line is a least squares fit to the points and the orange line is ν_6_ = ν_5_/√2 (Wilson’s rule). The circled points (red) were omitted from the least squares fit (see text).

This work has increased the number of systems for which ν_6_ is known by ∼30% (see Table S11). Thus it is interest to see if there are any systematic trends that can be exploited to estimate ν_6_, if the other modes are known. A trivial result is that ν_6_ always occurs below ν_4_ and ν_5_ and generally below ν_5_ because in almost all cases ν_4_ ≥ ν_5_. Table 2 shows that this is true for all the examples studied here^11^ and lists only a few cases where ν_5_ ≥ ν_4_. Figure 12 compares the transition energies for ν_5_ and ν_6_. It can be seen that there is a simple linear relationship between ν_5_ and ν_6_, enabling prediction of the latter. The four points that are circled are apparently anomalous and were omitted from the fit. Inclusion of these points does not change the fit parameters greatly but does result in an almost 10-fold increase in the residual sum of squares.

Inspection of the original literature shows that the assignment for [CrCl_6_]^3–^ as the Rb salt^55^ (in the upper left circle) is based on the presence of a shoulder on the low energy side of ν_4_. The structure of Rb_3_[CrCl_6_] is unknown, but the analogous Cs salt is orthorhombic,^56^*Pnnm*, with *Z* = 6. The presence of multiple ions in the primitive cell means that factor group splitting will be present, which may account for the shoulder on ν_4_. We note that there is no other example known where ν_5_ = ν_6_.

The three lower points are for TcF_6_, WF_6_, and MoF_6_ (from top to bottom).^57−60^ The assignment of ν_6_ here is based on the observation of the Raman allowed overtone 2ν_6_ in the gas phase. The Raman method is critically dependent on correct identification of the overtone, in systems such as these where there is significant population of excited states and hence, the presence of hot bands, this is not straightforward. For several systems (e.g. SF_6_, UF_6_) where ν_6_ has been determined by both this method and other techniques, the agreement is within a few wavenumbers (Table S11). However, ReF_6_ shows that this is not always the case and this is particularly so for MoF_6_, where values for ν_6_ of 117, 140, and 234 cm^–1^ have been proposed.^57,61,62^ A similar situation exists for WF_6_, where gas phase studies show ν_6_ at 127 cm^–1^, whereas it is observed at 147 cm^–1^ in the solid state.^61^ We note that ν_6_ at 140 and 147 cm^–1^, for MoF_6_ and WF_6_, respectively, would place them close to the least squares line. It is possible that there is a large shift between the gas phase and solid-state transition energies for these molecules (and by inference also for TcF_6_), but if so, it is very surprising that this is only true for ν_6_: ν_1_ to ν_5_ only show shifts of a few wavenumbers.

There have been a number of attempts to calculate ν_6_. The oldest method^63^ is based on a diagonal force field from which the relationship ν_6_ = ν_5_/√2 (Wilson’s rule) is obtained. This is plotted in Figure 12 as the solid orange line, and it can be seen that it is a reasonable approximation. However, the residual sum of squares is about triple that of the least squares fit (black line). A range of more sophisticated force fields have also been investigated,^64,65^ these generally do not predict ν_6_ any better than Wilson’s rule.

## Conclusions

In the present work, we have demonstrated that the addition of INS spectra to conventional infrared and Raman spectra enables unambiguous assignment of the internal M–X stretch and X–M–X bending modes by inspection. For cubic systems, the librational and translational modes are also readily assigned. For materials with a lower crystal symmetry, periodic-DFT provides a straightforward method for symmetry assignments, albeit it does require that the crystal structure of the low-temperature phase is known.

We have also shown that ν_6_ can be predicted with good accuracy, if ν_5_ is known (which is generally the case). If the apparently anomalous cases of TcF_6_, WF_6_, and MoF_6_ are omitted, the linear relation ν_6_ = 18 + 0.63ν_5_ predicts ν_6_ to better than 10% accuracy.

As has been mentioned, the INS spectra emphasize the enormous gains in sensitivity that have been achieved by modern instrumentation. The performance of VISION at SNS is so good that it is possible to obtain excellent quality spectra from only a few grams of material that has almost no cross section. This capability has obvious implications for the investigation of the inorganic halo perovskites that are of interest as photovoltaic materials. Phonons in these materials are important as they are involved in energy dissipation and may drive the structural phase changes that are commonly found in perovskites.

While INS spectroscopy may be considered a rare and exotic form of vibrational spectroscopy, in practice, it is readily accessible. The major facilities, ISIS (UK), SNS (USA), and Institut Laue Langevin (ILL, France) all operate a “mail-in” system, whereby all that is required is that the sample is “standard” (i.e., solid or liquid at room temperature and can be handled in air) and that sufficient information (usually just the composition) is provided to enable chemical and radiological safety assessments to be made.

## Supporting Information Available

INS spectra of cryolite over time; calculated dispersion curves and transition energies (with assignments) of K_2_[SiF_6_], Na_2_[SiF_6_], K_2_[TiF_6_], K[PF_6_], K_2_[PtCl_6_], K_2_[ReCl_6_], and K_2_[PtI_6_]; cif file for K[PF6] in phase III; structure of K_2_[PtI_6_]; correlation tables for Na_2_[SiF_6_] and K_2_[PtI_6_]; discussion and spectra of Na_3_[AlF_6_] and K_2_[PtCl_6_]; and multiwavelength Raman spectra of K_2_[PtBr_6_] (PDF)
