# Supplementary material for: Vibrational Spectroscopy of Hexahalo Complexes
Source: Inorg Chem. 2022 Apr 5;61(15):5844–54. doi: 10.1021/acs.inorgchem.2c00125 (PMC9171826; doi:10.1021/acs.inorgchem.2c00125)
Supplement: Supplementary file 1 — ic2c00125_si_001.pdf [file ic2c00125_si_001.pdf]

Electronic Supplementary Information

for:

The vibrational spectroscopy of hexahalo complexes

Stewart F. Parker,[1,2,*] Kenneth P. J. Williams,[3] Timothy Smith,[3] Anibal J. Ramirez-Cuesta[4] and Luke L. Daemen.[4]

[1]*ISIS Facility, STFC Rutherford Appleton Laboratory, Chilton, Didcot, OX11 0QX, UK*

[2]*School of Chemistry, University of Glasgow, Joseph Black Building, Glasgow, G12 8QQ, UK*

[3]*Renishaw plc, New Mills, Wotton-under-Edge, Gloucestershire. GL12 8JR, UK*

[4]*Neutron Science Directorate, Oak Ridge National Laboratory, Oak Ridge, TN 37831 USA*

⁎ Corresponding author. E-mail address: stewart.parker@stfc.ac.uk

Table of contents

Fig. S1 INS spectra of cryolite S-2
Fig. S2 Calculated dispersion curves of $K_2[SiF_6]$ in $Fm\bar{3}m$ S-3
Table S1 Calculated transition energies of $K_2[SiF_6]$ S-4
Fig. S3 Calculated dispersion curves of $Na_2[SiF_6]$ in *P*321 S-5
Table S2 Correlation table for $Na_2[SiF_6]$ S-6
Table S3 Calculated transition energies of $Na_2[SiF_6]$ S-7
Fig. S4 Calculated dispersion curves of $K_2[TiF_6]$ in $P\bar{3}m1$ S-9
Table S4 Calculated transition energies for $K_2[TiF_6]$ S-10
Table S5 cif file for $K[PF_6]$ in phase III S-11
Fig. S5 Calculated dispersion curves for the primitive cell of $K[PF_6]$ in phase III S-12
Table S6 Calculated transition energies for $K[PF_6]$ in phase III S-13
$Na_3[AlF_6]$ Discussion and **Fig. S6** spectra S-15
$K_2[PtCl_6]$ Discussion and **Fig. S7** spectra S-16
Table S7 Calculated transition energies for $K_2[PtCl_6]$ S-17
Fig. S8 Calculated dispersion curves of $K_2[PtCl_6]$ in $Fm\bar{3}m$ S-18
Fig. S9 Vibrational spectra of $K_2[ReCl_6]$ S-19
Fig. S10 Calculated dispersion curves of $K_2[ReCl_6]$ in $Fm\bar{3}m$ S-20
$K_2[PtBr_6]$ Multi-wavelength Raman spectra and **Fig. S11** S-21
Fig. S12 Structure of $K_2[PtI_6]$ in *Pmnc* and in $P2_1/c$ S-22
Fig. S13 Calculated dispersion curves of $K_2[PtI_6]$ in space group $P2_1/c$ S-23
Table S8 Calculated transition energies for $K_2[PtI_6]$ S-24
Table S9 Correlation table for $K_2[PtI_6]$ in *Pmnc* S-26
Table S10 Correlation table for $K_2[PtI_6]$ in $P2_1/c$ S-27
Table S11 Experimental transition energies of ν_5 and ν_6 for MF_6 molecules S-28
Table S12 Plane wave cut-offs and Monkhorst-Pack grids used for the complexes S-30
References S-31

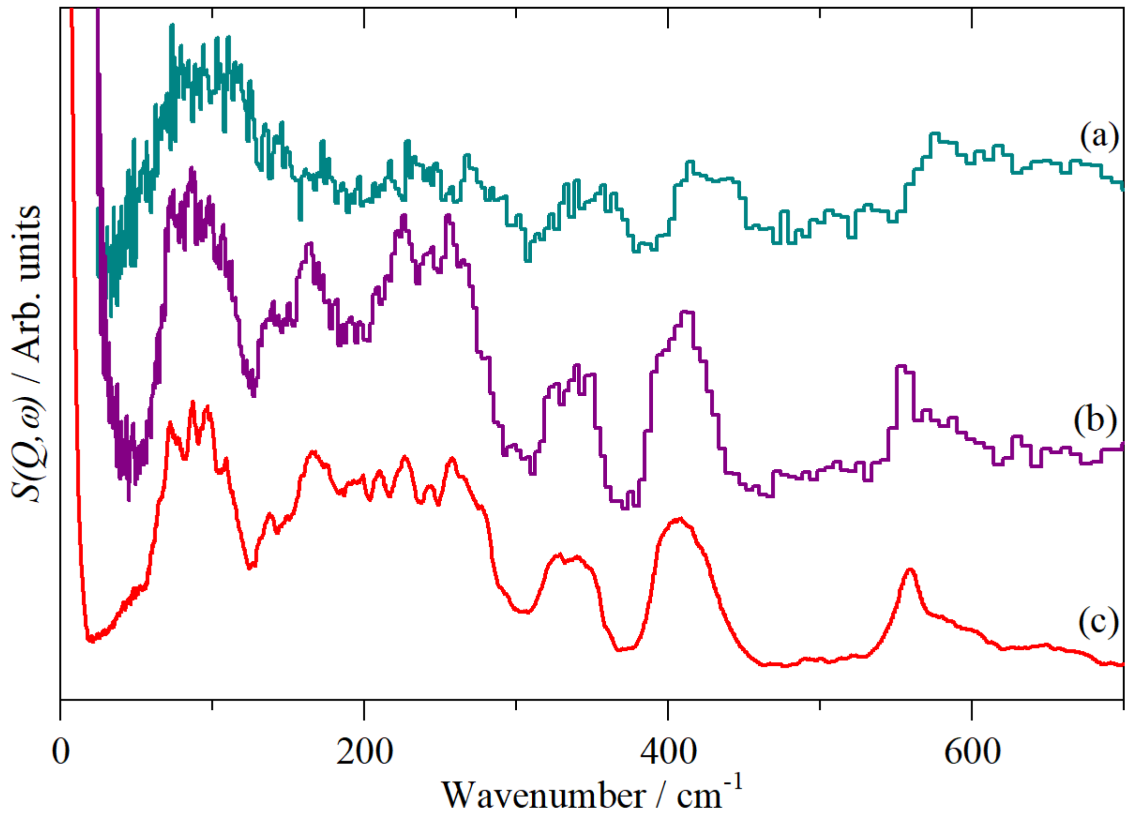

Fig. S1 INS spectra of cryolite, $Na_3[AlF_6]$ recorded with (a) TFXA at ISIS, (b)TOSCA (before the guide upgrade) and (c) VISION at SNS.

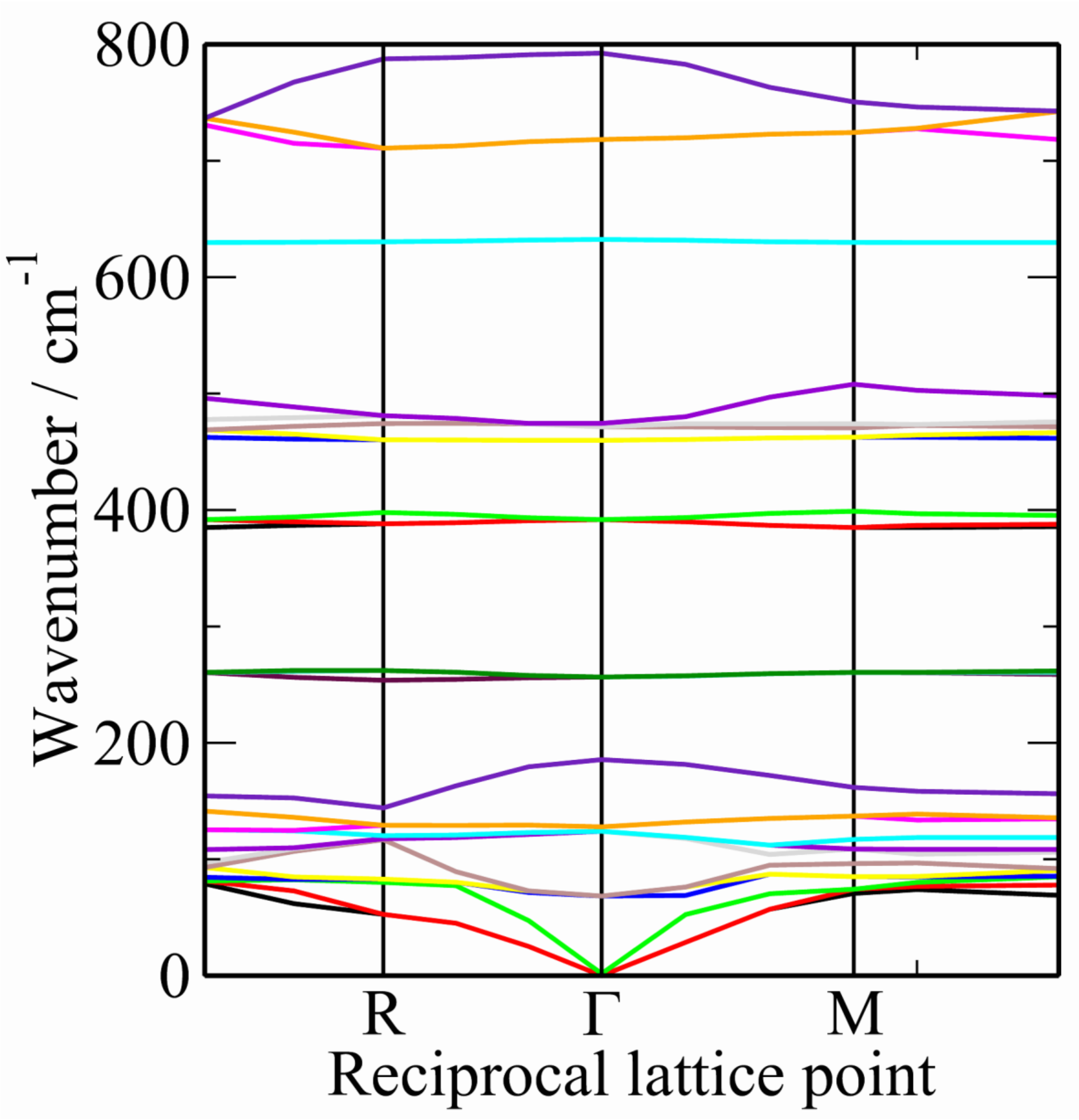

Fig. S2 Calculated dispersion curves of $K_2[SiF_6]$ in $Fm\bar{3}m$.

Table S1 Calculated transition energies of the primitive cell of $K_2[SiF_6]$ in $Fm\bar{3}m$ at the Brillouin zone Γ-point

Transition energy / cm^{-1}	Symmetry	Infrared active?	Infrared intensity km mol^{-1}	Raman active?	Raman intensity / $Å^{-4}$ amu^{-1}	Description
0	T_{1u}	N	0.00	N	0.00	Acoustic translation
0	T_{1u}	N	0.00	N	0.00	Acoustic translation
0	T_{1u}	N	0.00	N	0.00	Acoustic translation
68	T_{1g}	N	0.00	N	0.00	Libration
68	T_{1g}	N	0.00	N	0.00	Libration
68	T_{1g}	N	0.00	N	0.00	Libration
124	T_{2g}	N	0.00	Y	0.17	Optic translation
124	T_{2g}	N	0.00	Y	0.17	Optic translation
124	T_{2g}	N	0.00	Y	0.17	Optic translation
128	T_{1u}	Y	114.99	N	0.00	Optic translation
128	T_{1u}	Y	114.99	N	0.00	Optic translation
128	T_{1u}	Y	114.99	N	0.00	Optic translation
256	T_{2u}	N	0.00	N	0.00	ν_6
256	T_{2u}	N	0.00	N	0.00	ν_6
256	T_{2u}	N	0.00	N	0.00	ν_6
392	T_{2g}	N	0.00	Y	0.89	ν_5
392	T_{2g}	N	0.00	Y	0.89	ν_5
392	T_{2g}	N	0.00	Y	0.89	ν_5
460	T_{1u}	Y	82.15	N	0.00	ν_4
460	T_{1u}	Y	82.15	N	0.00	ν_4
460	T_{1u}	Y	82.15	N	0.00	ν_4
471	E_g	N	0.00	Y	0.33	ν_2
471	E_g	N	0.00	Y	0.33	ν_2
632	A_{1g}	N	0.00	Y	20.78	ν_1
718	T_{1u}	Y	499.83	N	0.00	ν_3
718	T_{1u}	Y	499.83	N	0.00	ν_3
718	T_{1u}	Y	499.83	N	0.00	ν_3

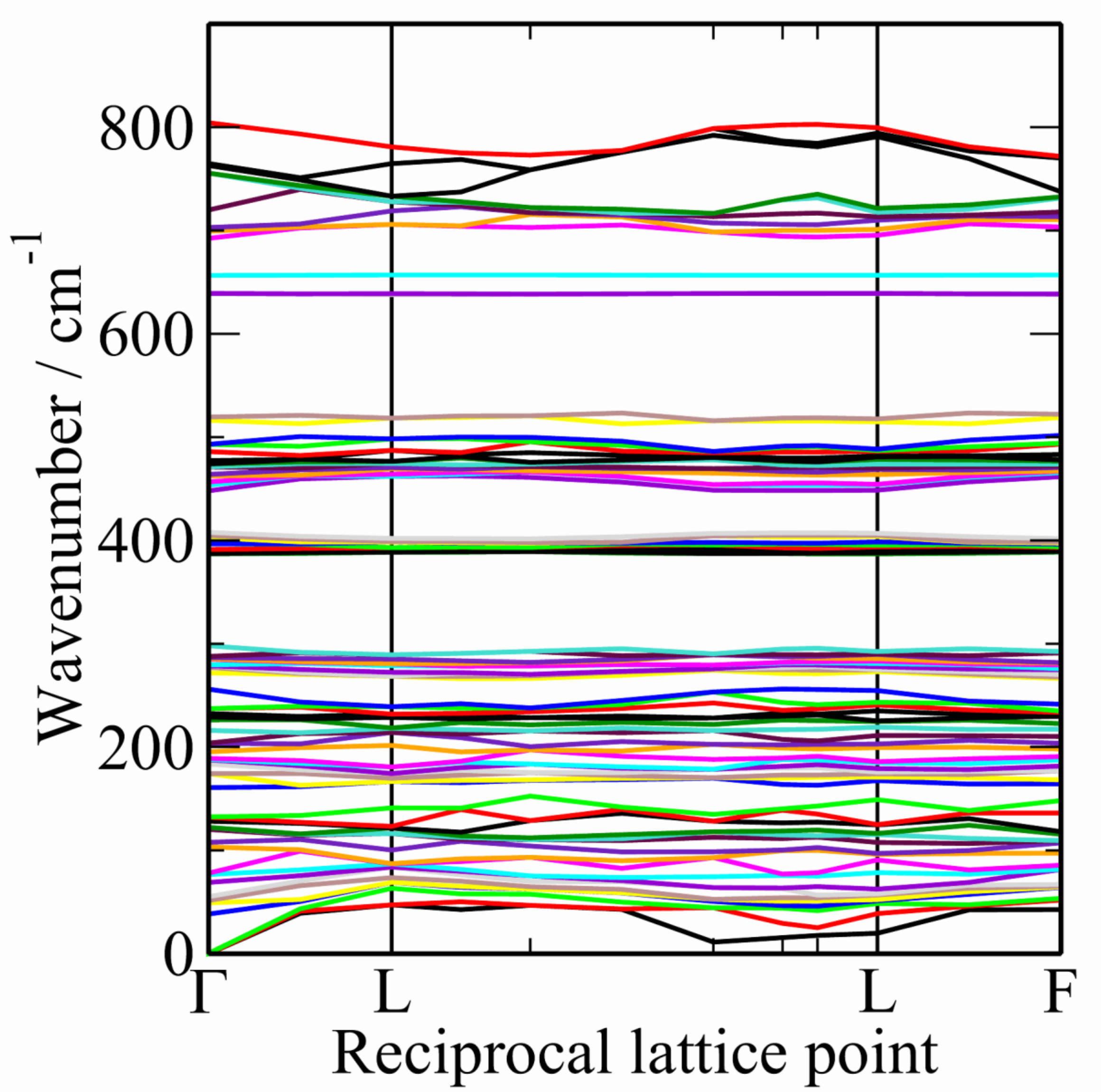

Fig. S3 Calculated dispersion curves of $Na_2[SiF_6]$ in *P*321.

Table S2 Correlation table for $Na_2[SiF_6]$.

**Ion**	***n***	**Free ion**		**Crystal**		**Translations**		**Librations**		**Intramolecular**		**Factor group** D_3	**Total** [1]
		**Sym.** [2]	**Rep.**	**Site**[3]	**W**[4]	**Rep.**	**No.**	**Rep.**	**No.**	**Rep.**	**No.**		
Na1	3			C_2	e	$A + 2B$	9					$A_1 + 2A_2$	$3\ (A_1 + 2A_2)$
Na2	3			C_2	f	$A + 2B$	9					$A_1 + 2A_2$	$3\ (A_1 + 2A_2)$
SiF_6	1	O_h	T_{1u}	D_3	a	$A_2 + E$	3					$A_2 + E$	$(A_2 + E)$
	1	O_h	T_{1g}	D_3	a			$A_2 + E$	3			$A_2 + E$	$(A_2 + E)$
	1	O_h	A_{1g} (ν_1)	D_3	a					A_1	1	A_1	(A_1)
	1	O_h	E_g (ν_2)	D_3	a					E	2	E	(E)
	1	O_h	T_{1u} (ν_3)	D_3	a					$A_2 + E$	3	$A_2 + E$	$(A_2 + E)$
	1	O_h	T_{1u} (ν_4)	D_3	a					$A_2 + E$	3	$A_2 + E$	$(A_2 + E)$
	1	O_h	T_{2g} (ν_5)	D_3	a					$A_1 + E$	3	$A_1 + E$	$(A_1 + E)$
	1	O_h	T_{2u} (ν_6)	D_3	a					$A_1 + E$	3	$A_1 + E$	$(A_1 + E)$
SiF_6	2	O_h	T_{1u}	C_3	d	$A + E$	6					$A_1 + A_2 + 2E$	$(A_1 + A_2 + 2E)$
	2	O_h	T_{1g}	C_3	d			$A + E$	6			$A_1 + A_2 + 2E$	$(A_1 + A_2 + 2E)$
	2	O_h	A_{1g} (ν_1)	C_3	d					A	2	$A_1 + A_2$	$(A_1 + A_2)$
	2	O_h	E_g (ν_2)	C_3	d					E	4	$2E$	$(2E)$
	2	O_h	T_{1u} (ν_3)	C_3	d					$A + E$	6	$A_1 + A_2 + 2E$	$(A_1 + A_2 + 2E)$
	2	O_h	T_{1u} (ν_4)	C_3	d					$A + E$	6	$A_1 + A_2 + 2E$	$(A_1 + A_2 + 2E)$
	2	O_h	T_{2g} (ν_5)	C_3	d					$A + E$	6	$A_1 + A_2 + 2E$	$(A_1 + A_2 + 2E)$
	2	O_h	T_{2u} (ν_6)	C_3	d					$A + E$	6	$A_1 + A_2 + 2E$	$(A_1 + A_2 + 2E)$

[1] Total is the product of the column "No." (where No. is the total number of vibrations of that type in the primitive cell) and the factor group.
[2] Sym. = symmetry, Rep. = irreducible representation of the point group, No. = number.
[3] Symmetry of the site occupied by the ion in the crystal.
[4]Wyckoff site.

Table S3 Calculated transition energies of $Na_2[SiF_6]$ in *P*321 at the Brillouin zone Γ-point.

Transition energy / cm^{-1}	Symmetry	Infrared active?	Infrared intensity km mol^{-1}	Raman active?	Description
0	A_2	N	0.00	N	Acoustic translation
0	E	N	0.00	N	Acoustic translation
0	E	N	0.00	N	Acoustic translation
38	A_2	Y	4.74	N	Si1 Libration
49	E	Y	7.55	Y	Si1 Libration
49	E	Y	7.55	Y	Si1 Libration
55	A_1	N	0.00	Y	Si2 Libration
69	A_2	Y	78.35	N	Libration
77	E	Y	6.58	Y	Optic translation + Libration
77	E	Y	6.58	Y	Optic translation + Libration
103	E	Y	26.48	Y	Optic translation
103	E	Y	26.48	Y	Optic translation
120	A_2	Y	10.47	N	Optic translation + Libration
122	E	Y	0.18	Y	Optic translation
122	E	Y	0.18	Y	Optic translation
128	A_2	N	0.00	Y	Optic translation + Libration
132	E	Y	3.17	Y	Optic translation + Libration
132	E	Y	3.17	Y	Optic translation + Libration
161	A_2	Y	1.23	N	Optic translation
174	E	Y	5.62	Y	Optic translation
174	E	Y	5.62	Y	Optic translation
184	E	Y	123.04	Y	Optic translation
184	E	Y	123.04	Y	Optic translation
188	E	Y	11.37	Y	Optic translation
188	E	Y	11.37	Y	Optic translation
189	A_1	N	0.00	Y	Optic translation + Libration
204	E	Y	0.01	Y	Optic translation
204	E	Y	0.01	Y	Optic translation
216	A_2	Y	73.47	N	Optic translation
226	E	Y	136.10	Y	Optic translation
226	E	Y	136.10	Y	Optic translation
229	A_1	N	0.00	Y	Optic translation
237	E	Y	78.73	Y	Optic translation
237	E	Y	78.73	Y	Optic translation
237	A_2	Y	115.82	N	Optic translation
272	A_2	Y	31.95	N	Optic translation
277	A_1	N	0.00	Y	ν_6
279	E	Y	0.07	Y	ν_6

279	E	Y	0.07	Y	ν_6
280	E	Y	62.93	Y	ν_6
280	E	Y	62.93	Y	ν_6
285	A_1	N	0.00	Y	ν_6
287	A_2	Y	65.11	N	ν_6
288	E	Y	16.24	Y	ν_6
288	E	Y	16.24	Y	ν_6
387	E	Y	3.46	Y	ν_5
387	E	Y	3.46	Y	ν_5
391	E	Y	0.41	Y	ν_5
391	E	Y	0.41	Y	ν_5
397	E	Y	0.01	Y	ν_5
397	E	Y	0.01	Y	ν_5
403	A_2	Y	1.32	N	ν_5
405	A_1	N	0.00	Y	ν_5
408	A_1	N	0.00	Y	ν_5
448	A_2	Y	122.20	N	ν_4
454	A_1	N	0.00	Y	ν_4
457	A_2	Y	68.89	N	ν_4
463	E	Y	33.65	Y	ν_2
463	E	Y	33.65	Y	ν_2
471	E	Y	2.31	Y	ν_2
471	E	Y	2.31	Y	ν_2
474	E	Y	0.06	Y	ν_2
474	E	Y	0.06	Y	ν_2
477	E	Y	131.88	Y	ν_4
477	E	Y	131.88	Y	ν_4
493	E	Y	0.60	Y	ν_4
493	E	Y	0.60	Y	ν_4
516	E	Y	43.55	Y	ν_4
516	E	Y	43.55	Y	ν_4
639	A_1	N	0.00	Y	ν_1
639	A_2	Y	10.71	N	ν_1
657	A_1	N	0.00	Y	ν_1
692	A_1	N	0.00	Y	ν_3
699	A_2	Y	1635.05	N	ν_3
703	E	Y	1604.39	Y	ν_3
703	E	Y	1604.39	Y	ν_3
720	A_2	Y	0.06	N	ν_3
756	E	Y	0.00	Y	ν_3
756	E	Y	0.00	Y	ν_3
765	E	Y	21.33	Y	ν_3
765	E	Y	21.33	Y	ν_3

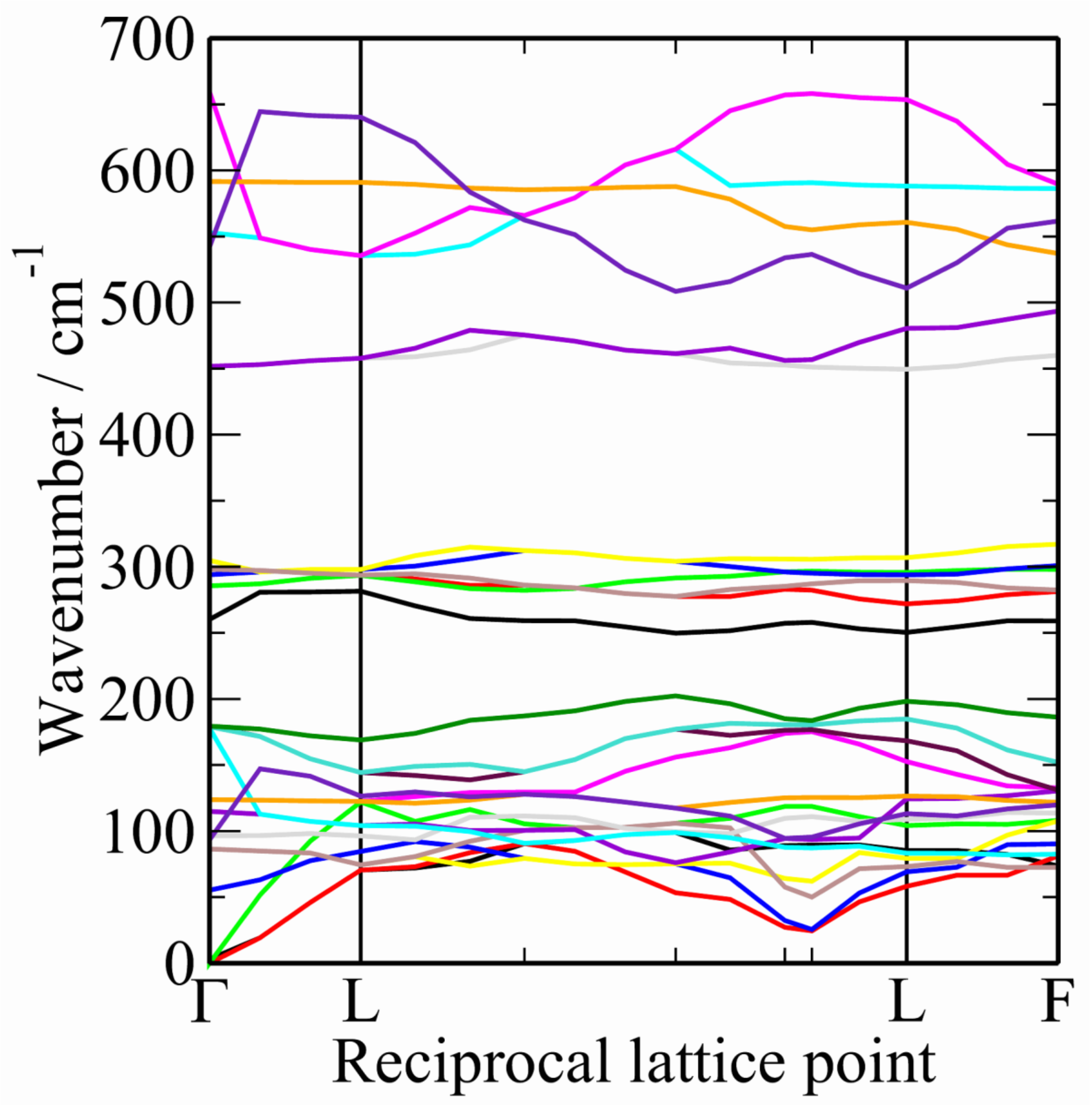

Fig. S4 Calculated dispersion curves of $K_2[TiF_6]$ in $P\bar{3}m1$.

Table S4 Calculated transition energies for $K_2[TiF_6]$ in space group $P\bar{3}m1$ at the Brillouin zone Γ-point.

Transition energy / cm^{-1}	Symmetry	Infrared active?	Infrared intensity km mol^{-1}	Raman active?	Description
0	E_u	N	0.00	N	Acoustic translation
0	E_u	N	0.00	N	Acoustic translation
0	A_{2u}	N	0.00	N	Acoustic translation
55	A_{2g}	N	0.00	N	Libration
86	E_g	N	0.00	Y	Libration
86	E_g	N	0.00	Y	Libration
93	A_{2u}	Y	128.08	N	Optic translation
97	A_{1g}	N	0.00	Y	K^+ translation
115	E_u	Y	172.54	N	Optic translation
115	E_u	Y	172.54	N	Optic translation
124	E_g	N	0.00	Y	K^+ translation
124	E_g	N	0.00	Y	K^+ translation
179	E_u	Y	0.04	N	ν_6
179	E_u	Y	0.04	N	ν_6
179	A_{1u}	N	0.00	N	ν_6
260	A_{2u}	Y	78.57	N	ν_5
286	E_g	N	0.00	Y	ν_5
286	E_g	N	0.00	Y	ν_5
294	E_u	Y	45.14	N	ν_4
294	E_u	Y	45.14	N	ν_4
298	A_{1g}	N	0.00	Y	ν_4
452	E_g	N	0.00	Y	ν_2
452	E_g	N	0.00	Y	ν_2
542	A_{2u}	Y	648.00	N	ν_3
553	E_u	Y	692.98	N	ν_3
553	E_u	Y	692.98	N	ν_3
592	A_{1g}	N	0.00	Y	ν_1

Table S5 cif file for $K[PF_6]$ in phase III. (Reproduced from [1]).

```
data_KPF6_Cockcroft_Phase_III_C2c
_symmetry_space_group_name_H-M    'C2/C'
_symmetry_Int_Tables_number       15
_symmetry_cell_setting            monoclinic
loop_
_symmetry_equiv_pos_as_xyz
  x,y,z
  -x,y,-z+1/2
  -x,-y,-z
  x,-y,z+1/2
  x+1/2,y+1/2,z
  -x+1/2,y+1/2,-z+1/2
  -x+1/2,-y+1/2,-z
  x+1/2,-y+1/2,z+1/2
_cell_length_a                    9.4230
_cell_length_b                    4.9058
_cell_length_c                    9.4660
_cell_angle_alpha                 90.0000
_cell_angle_beta                  103.3500
_cell_angle_gamma                 90.0000
loop_
_atom_site_label
_atom_site_type_symbol
_atom_site_fract_x
_atom_site_fract_y
_atom_site_fract_z
_atom_site_U_iso_or_equiv
_atom_site_adp_type
_atom_site_occupancy
F1   F  f  0.22113  -0.12093   0.64643   0.00000  Uiso   1.00
F2   F  f  0.32621   0.02847   0.46874   0.00000  Uiso   1.00
F3   F  f  0.09529  -0.13756   0.40961   0.00000  Uiso   1.00
P    P  c  0.25000  -0.25000   0.50000   0.00000  Uiso   1.00
K    K  e  0.50000  -0.15700   1.25000   0.00000  Uiso   1.00
loop_
_geom_bond_atom_site_label_1
_geom_bond_atom_site_label_2
_geom_bond_distance
_geom_bond_site_symmetry_2
_ccdc_geom_bond_type
F1   P    1.603  .     S
F2   P    1.602  .     S
F3   P    1.608  .     S
P    F2   1.602  7_546 S
P    F1   1.603  7_546 S
P    F3   1.608  7_546 S
```

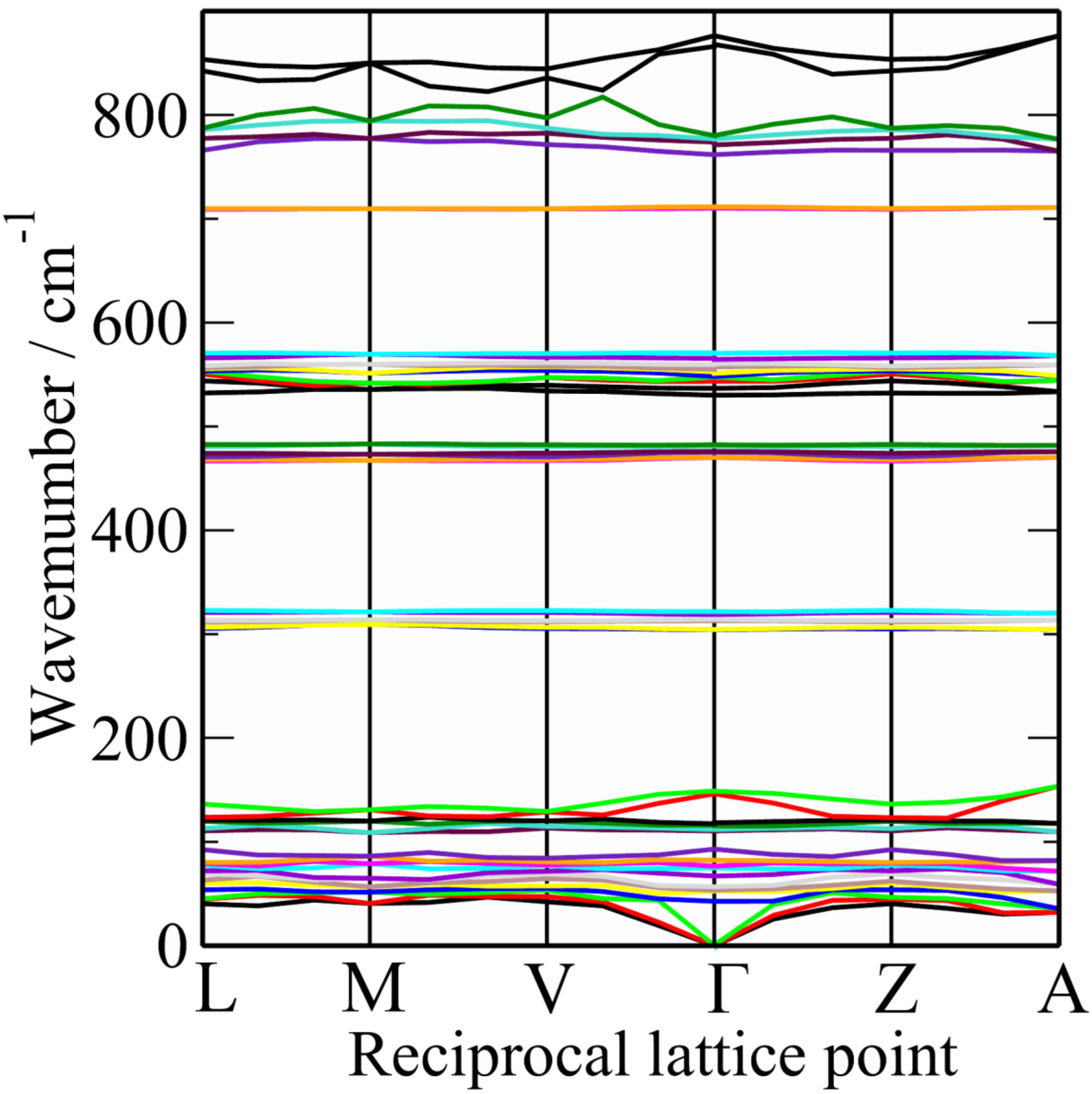

Fig. S5 Calculated dispersion curves for the primitive cell of K[PF_6] in phase III, space group *C*2/c.

Table S6 Calculated transition energies for the primitive cell of $K[PF_6]$ in phase III, space group *C*2/c at the Brillouin zone Γ-point.

Transition energy / cm^{-1}	Symmetry	Infrared active?	Infrared intensity km mol^{-1}	Raman active?	Description
0	B_u	N	0.00	N	Acoustic translation
0	A_u	N	0.00	N	Acoustic translation
0	B_u	N	0.00	N	Acoustic translation
43	B_g	N	0.00	Y	Libration
52	A_g	N	0.00	Y	Libration
53	B_u	Y	2.49	N	Translation
56	A_u	Y	1.77	N	Translation
67	A_g	N	0.00	Y	Libration
75	B_g	N	0.00	Y	Libration
77	B_g	N	0.00	Y	Libration
82	A_g	N	0.00	Y	Libration
93	A_u	Y	0.38	N	Translation
111	A_g	N	0.00	Y	Translation
112	B_g	N	0.00	Y	Translation
113	A_u	Y	84.78	N	Translation
116	B_u	Y	116.37	N	Translation
119	B_u	Y	114.62	N	Translation
146	B_g	N	0.00	Y	Translation
304	A_u	Y	0.01	N	ν_6
305	B_u	Y	1.07	N	ν_6
313	A_u	Y	0.11	N	ν_6
314	B_u	Y	0.36	N	ν_6
319	A_u	Y	0.30	N	ν_6
322	B_u	Y	1.01	N	ν_6
470	B_g	N	0.00	Y	ν_5
470	A_g	N	0.00	Y	ν_5
475	B_g	N	0.00	Y	ν_5
476	A_g	N	0.00	Y	ν_5
481	A_g	N	0.00	Y	ν_5
482	B_g	N	0.00	Y	ν_5
530	B_g	N	0.00	Y	ν_2
537	A_g	N	0.00	Y	ν_2
543	A_g	N	0.00	Y	ν_2
548	B_g	N	0.00	Y	ν_2
548	B_u	Y	20.80	N	ν_4
548	A_u	Y	108.25	N	ν_4
554	B_u	Y	132.85	N	ν_4
559	A_u	Y	11.56	N	ν_4
564	B_u	Y	97.86	N	ν_4
568	A_u	Y	24.14	N	ν_4
710	B_g	N	0.00	Y	ν_1
712	A_g	N	0.00	Y	ν_1
762	B_u	Y	848.66	N	ν_3
762	A_u	Y	1171.00	N	ν_3

775	B_u	Y	958.50	N	ν_3
777	B_u	Y	690.47	N	ν_3
780	A_u	Y	34.23	N	ν_3
876	A_u	Y	0.33	N	ν_3

$Na_3[AlF_6]$. This material is better known as the mineral cryolite and the spectra (including the INS) have been comprehensively assigned elsewhere [2]. Fig. S6 shows the spectra and the comparison with the DFT calculation. The spectra are assigned as: the strong mode at 554 cm^{-1} in the Raman spectrum is ν_1 and the intense, broad mode at 560 cm^{-1} in the infrared spectrum is ν_3. The two weaker modes at 396 and 344 cm^{-1} in the Raman spectrum are ν_2 and ν_5 respectively. In the infrared spectrum the mode at 396 cm^{-1} is assigned as ν_4. This is clear from the INS spectrum, which shows a mode at 407 cm^{-1}, that is much stronger than ν_5, consistent with it being the unresolved sum of the two modes. The optically silent mode ν_6 is not obvious; the calculations show it to occur at 250 - 280 cm^{-1} at the high energy edge of the lattice modes (0 – 300 cm^{-1}).

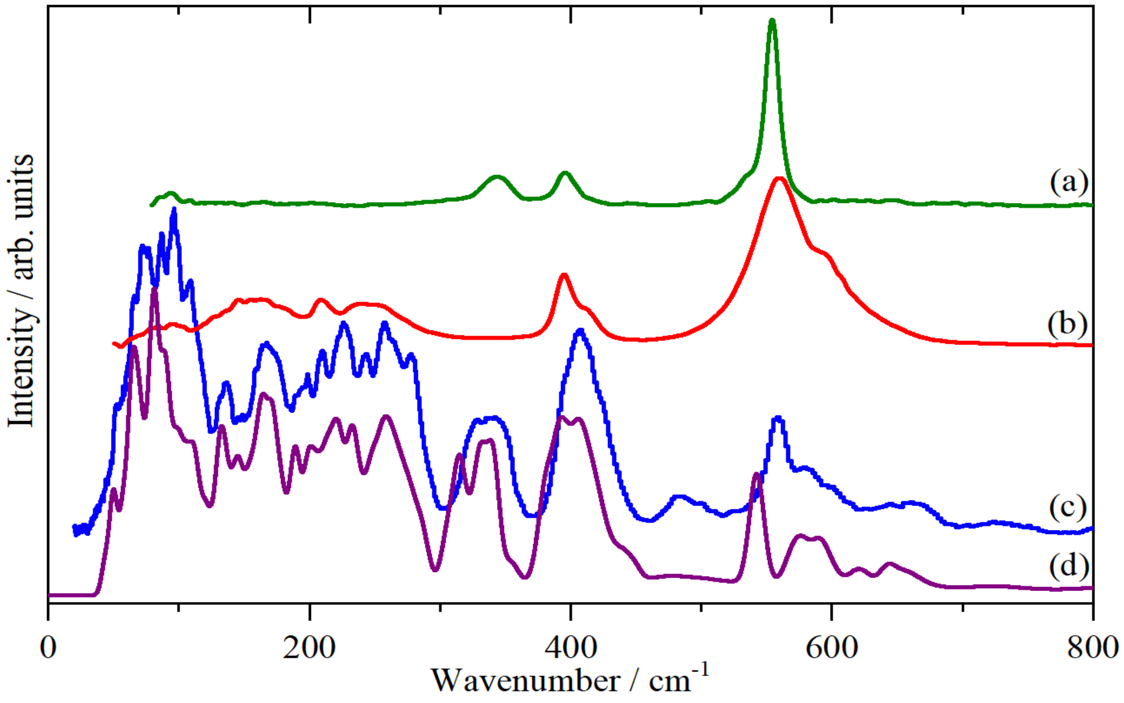

Fig. S6 Vibrational spectra of $Na_3[AlF_6]$ (cryolite) [2]: (a) Raman at room temperature (1064 nm excitation), (b) infrared at room temperature, (c) INS at 5 K recorded on VISION and (d) generated from the CASTEP output of the calculation in the space group $P2_1/n$. Adapted from ref. [2] under the Creative Commons Attribution 3.0 Unported licence (CC BY 3.0).

$K_2[PtCl_6]$. The structure of $K_2[PtCl_6]$ has been studied on multiple occasions: the Inorganic Crystal Structure Database (ICSD) [3] lists 27 determinations. This is perhaps not surprising as $K_2[PtCl_6]$ is the archetype of this structural form. Temperature dependent measurements show that the $Fm\bar{3}m$ symmetry is maintained down to at least 8 K [4].

The spectroscopy of $K_2[PtCl_6]$ has been comprehensively investigated [5-11] and our infrared and Raman spectra, Figs. S7a,b are in agreement with the literature. Our DFT calculations, Figs. S7d and S8, confirm the previous assignments [11], see Tables 2 and S6.

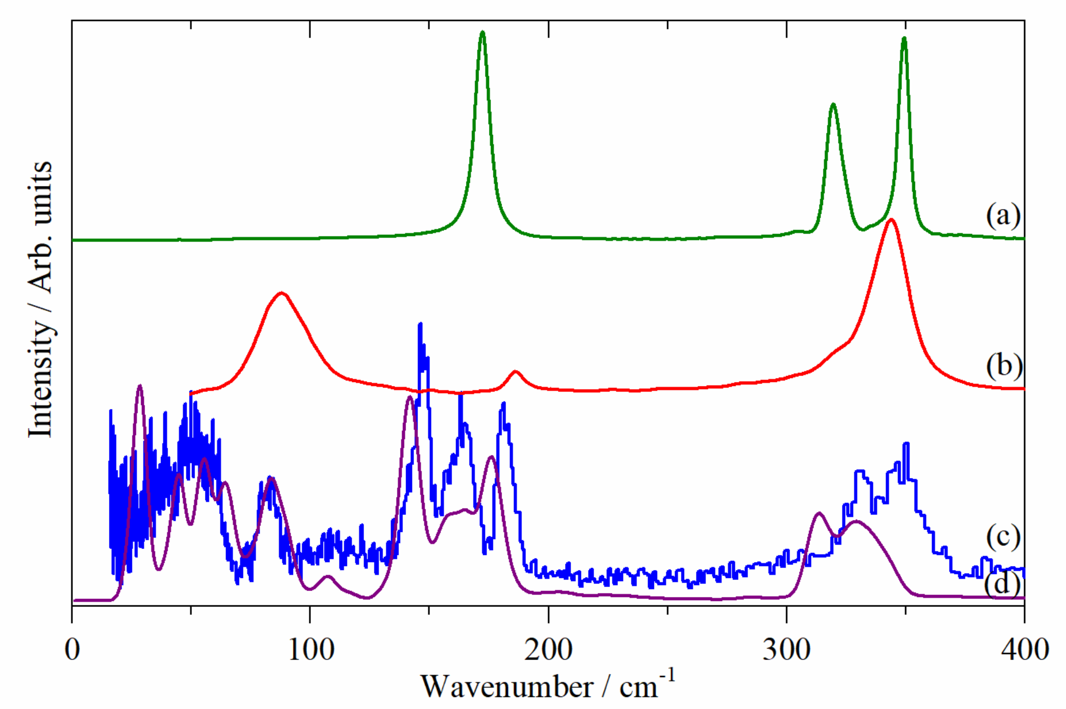

Fig. S7 Vibrational spectra of $K_2[PtCl_6]$: (a) Raman at room temperature (1064 nm excitation), (b) infrared (as a polyethylene disc) at room temperature, (c) INS at 5 K recorded on TFXA and (d) generated from the CASTEP output of the calculation in the space group $Fm\bar{3}m$.

Table S7 Calculated transition energies for the primitive cell of $K_2[PtCl_6]$ in space group in $Fm\bar{3}m$ at the Brillouin zone Γ-point.

Transition energy / cm^{-1}	Symmetry	Infrared active?	Infrared intensity km mol^{-1}	Raman active?	Raman intensity / $Å^{-4}$ amu^{-1}	Description
0	T_{1u}	N	0.00	N	0.00	Acoustic translation
0	T_{1u}	N	0.00	N	0.00	Acoustic translation
0	T_{1u}	N	0.00	N	0.00	Acoustic translation
55	T_{1g}	N	0.00	N	0.00	Libration
55	T_{1g}	N	0.00	N	0.00	Libration
55	T_{1g}	N	0.00	N	0.00	Libration
80	T_{1u}	Y	114.99	N	0.00	Optic translation
80	T_{1u}	Y	114.99	N	0.00	Optic translation
80	T_{1u}	Y	114.99	N	0.00	Optic translation
82	T_{2g}	N	0.00	Y	0.17	Optic translation
82	T_{2g}	N	0.00	Y	0.17	Optic translation
82	T_{2g}	N	0.00	Y	0.17	Optic translation
140	T_{2u}	N	0.00	N	0.00	ν_6
140	T_{2u}	N	0.00	N	0.00	ν_6
140	T_{2u}	N	0.00	N	0.00	ν_6
167	T_{2g}	N	0.00	Y	0.89	ν_5
167	T_{2g}	N	0.00	Y	0.89	ν_5
167	T_{2g}	N	0.00	Y	0.89	ν_5
188	T_{1u}	Y	82.15	N	0.00	ν_4
188	T_{1u}	Y	82.15	N	0.00	ν_4
188	T_{1u}	Y	82.15	N	0.00	ν_4
295	E_g	N	0.00	Y	0.33	ν_2
295	E_g	N	0.00	Y	0.33	ν_2
315	A_{1g}	N	0.00	Y	20.78	ν_1
318	T_{1u}	Y	499.83	N	0.00	ν_3
318	T_{1u}	Y	499.83	N	0.00	ν_3
318	T_{1u}	Y	499.83	N	0.00	ν_3

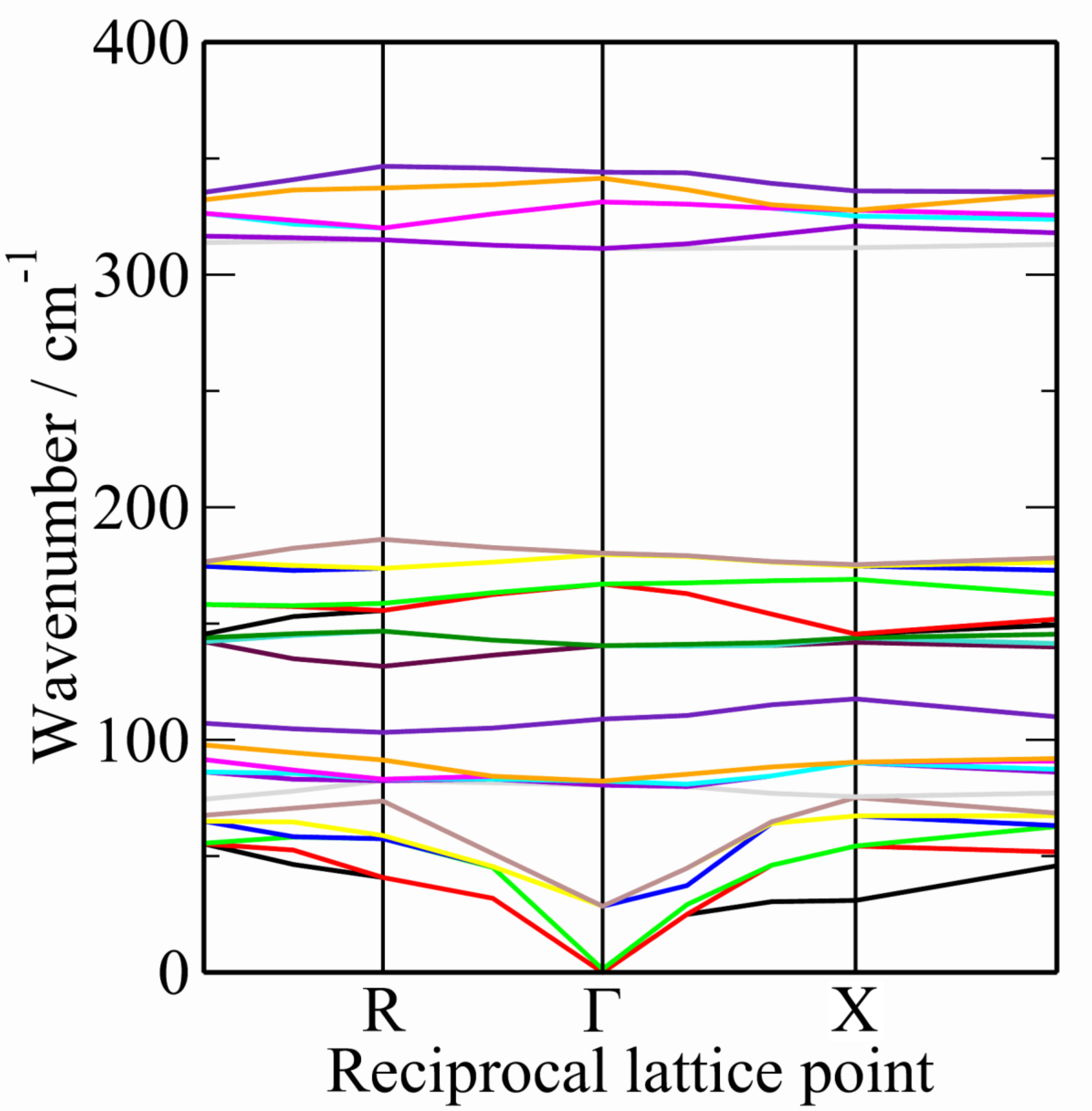

Fig. S8 Calculated dispersion curves of $K_2[PtCl_6]$ in $Fm\bar{3}m$.

$K_2[ReCl_6]$.

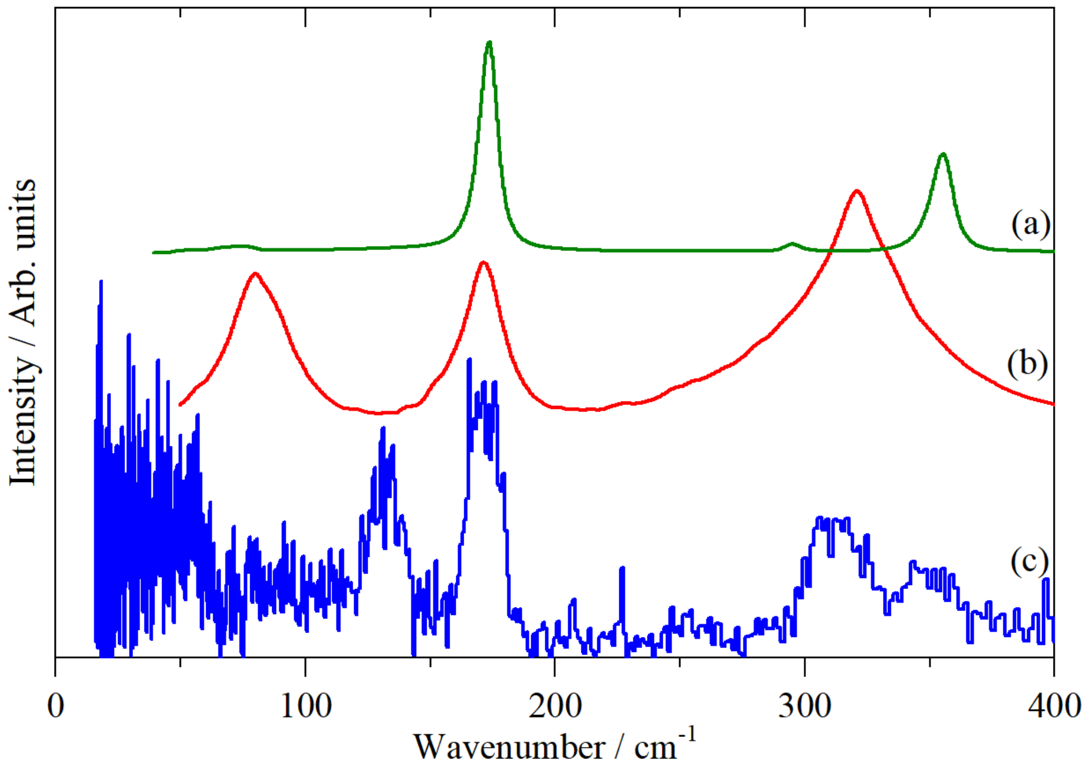

Fig. S9 Vibrational spectra of $K_2[ReCl_6]$: (a) Raman (532 nm excitation), (b) infrared and (c) INS recorded on TFXA. (a) and (b) are at room temperature and (c) at 5 K. The Raman spectrum shows ν_1, ν_2 and ν_5 at 355, 295 and 174 cm^{-1} respectively, the infrared shows ν_3 and ν_4 at 321 and 172 cm^{-1} respectively and the INS shows ν_6 at 132 cm^{-1}.

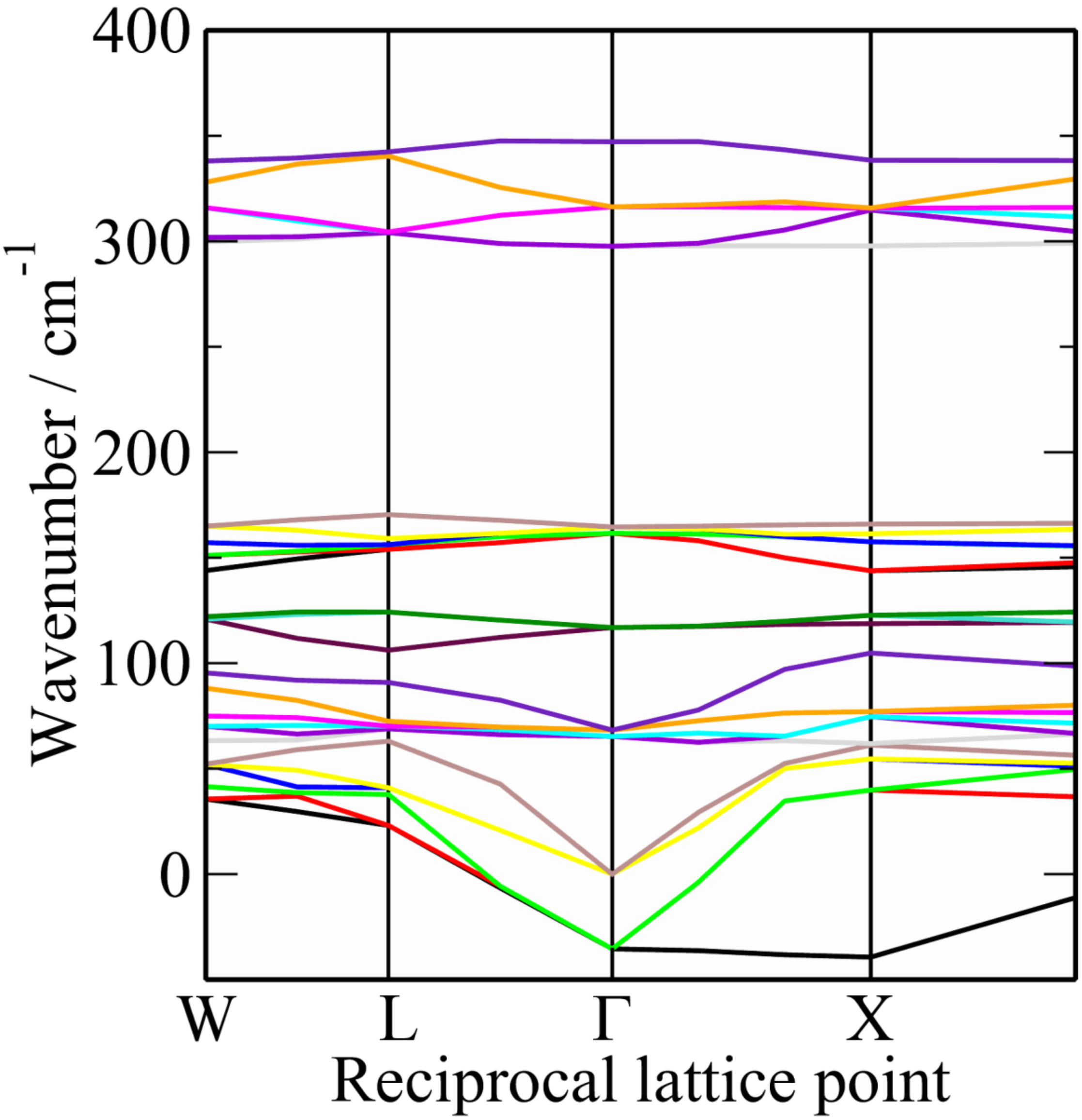

Fig. S10 Calculated dispersion curves of $K_2[ReCl_6]$ in $Fm\bar{3}m$.

$K_2[PtBr_6]$. $K_2[PtBr_6]$ is highly coloured (it is very dark red, almost black). The ammonium salt is known [12] to show an overtone progression up to $7\nu_1$ with 325 nm excitation. Using modern instrumentation, we were interested to see if it was possible to observe resonance Raman spectra involving some of the other modes, both as a means of assessing the anharmonicity present and as an alternative means of viewing inactive modes. Unfortunately, as Fig. S11 shows, we were only able to observe the first overtones of ν_1 and ν_2 and the binary combination $\nu_1 + \nu_2$ (386 $cm^{-1} = 2\nu_2$, 402 $cm^{-1} = \nu_1 + \nu_2$, 421 $cm^{-1} = 2\nu_1$).

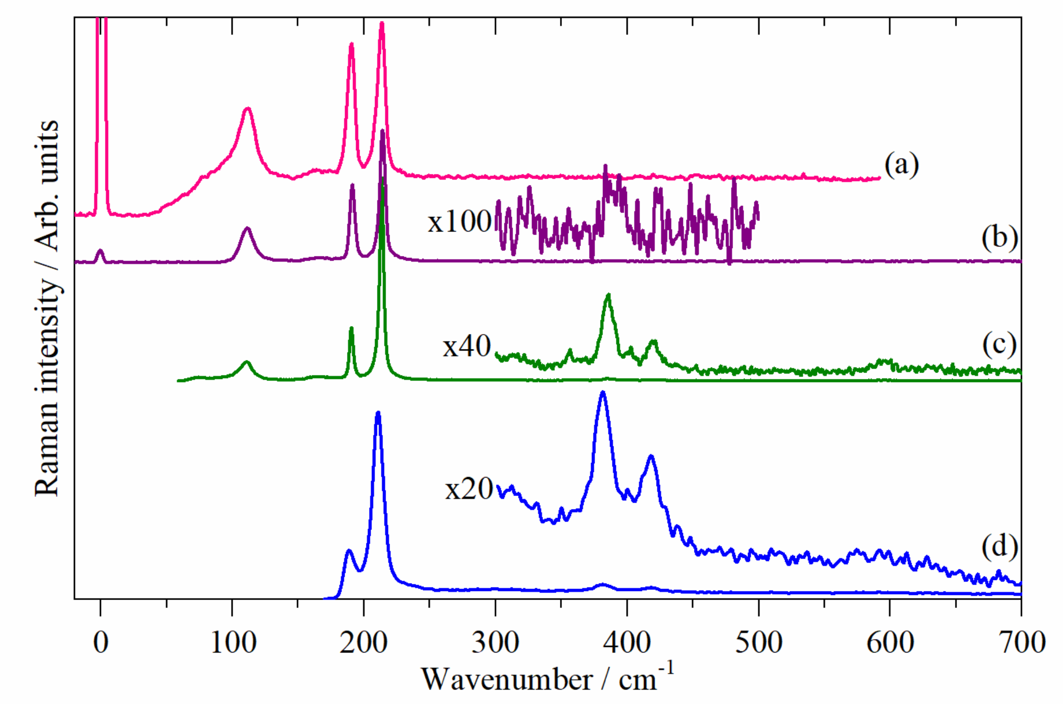

Fig. S11 Raman spectra of $K_2[PtBr_6]$ in the cubic phase as a function of excitation wavelength: (a) 785 nm, (b) 633 nm, (c) 532 nm and (d) 405 nm.

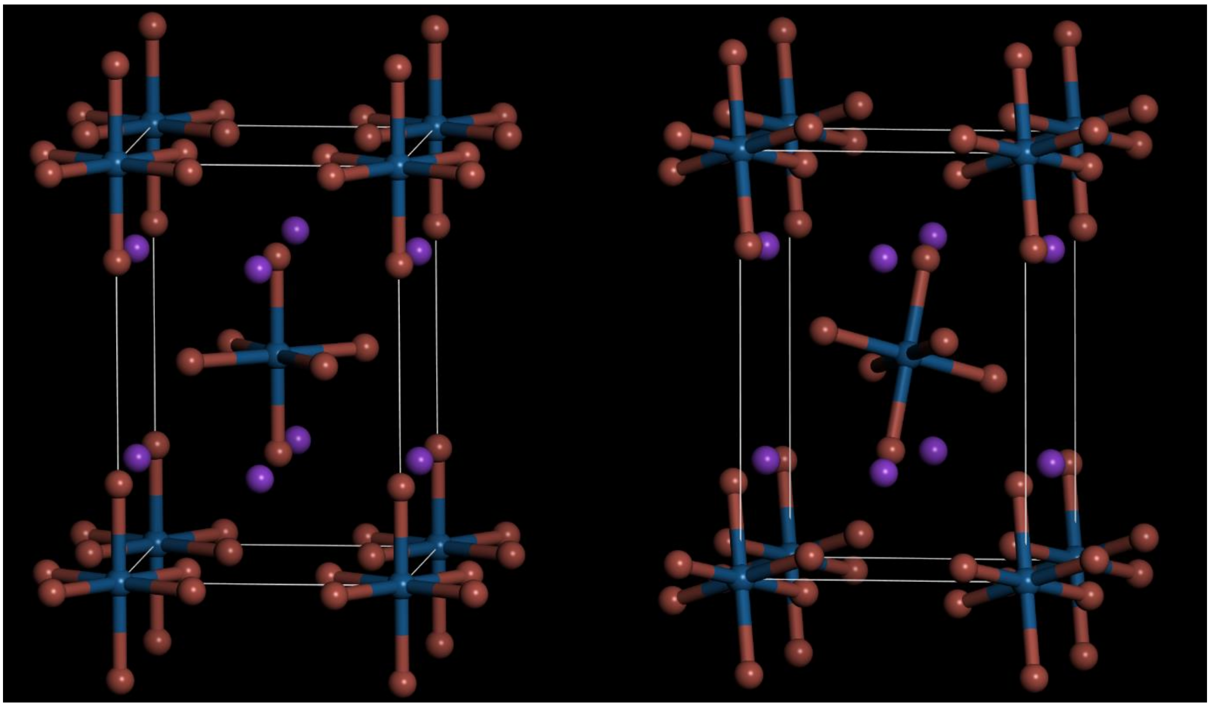

Fig. S12 Structure of $K_2[PtI_6]$ in *Pmnc* (left) and after tilting the PtI_6 octahedra to generate $P2_1/c$ (right).

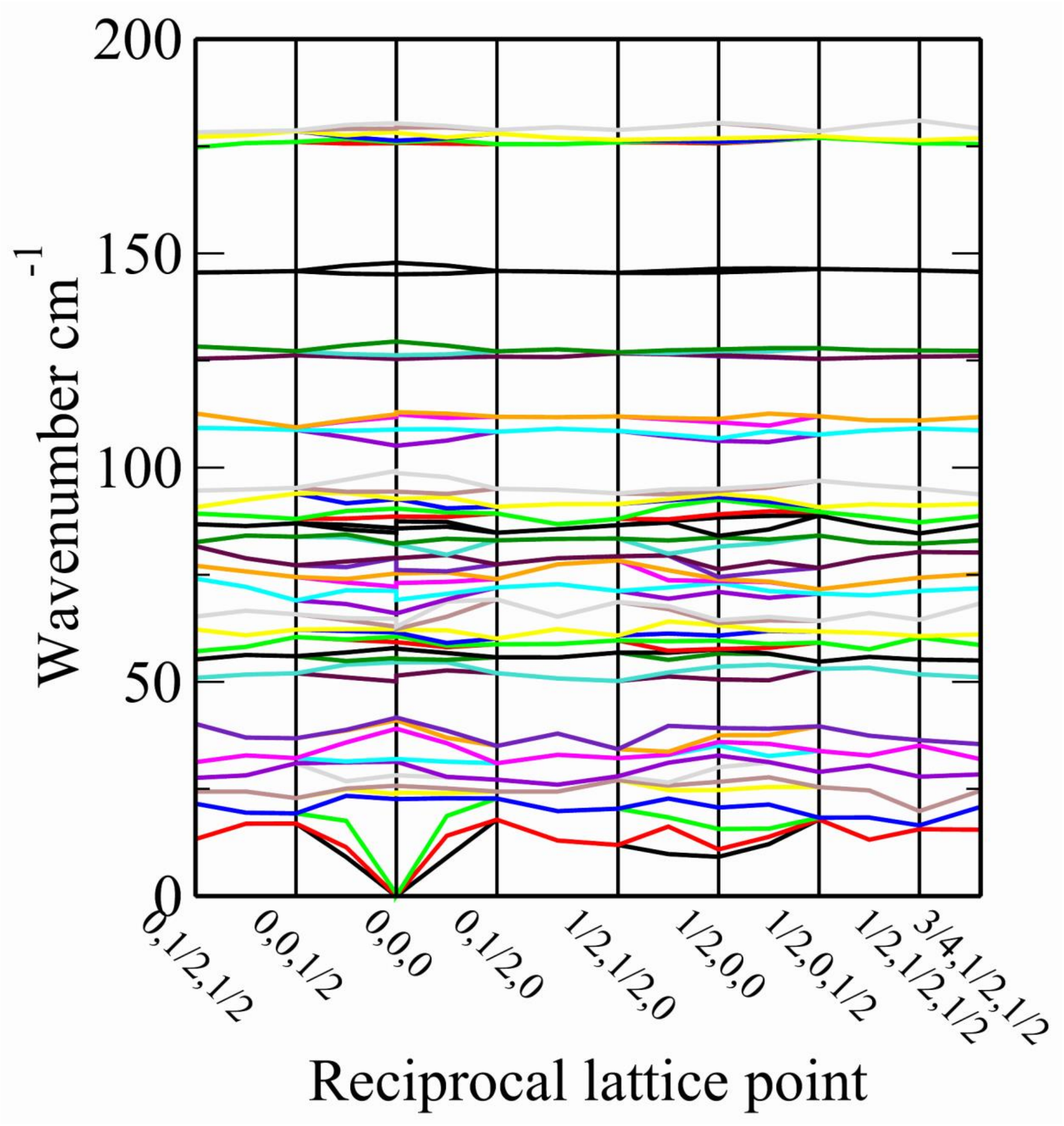

Fig. S13 Calculated dispersion curves of $K_2[PtI_6]$ in space group $P2_1/c$.

Table S8 Calculated transition energies for the primitive cell of $K_2[PtI_6]$ in space group $P2_1/c$ at the Brillouin zone Γ-point.

Transition energy / cm^{-1}	Symmetry	Infrared active?	Infrared intensity km mol^{-1}	Raman active?	Raman intensity $Å^4$ amu^{-1}	Description
0	B_u	N	0.00	N	0.00	Acoustic translation
0	A_u	N	0.00	N	0.00	Acoustic translation
0	B_u	N	0.00	N	0.00	Acoustic translation
23	A_g	N	0.00	Y	41.22	Libration
24	A_u	Y	2.02	N	0.00	PtI_6 translation
26	B_u	Y	2.96	N	0.00	PtI_6 translation
28	B_g	N	0.00	Y	6.60	Libration
31	A_g	N	0.00	Y	53.24	Libration
32	B_g	N	0.00	Y	7.45	Libration
39	B_g	N	0.00	Y	7.36	Libration
41	A_g	N	0.00	Y	85.30	Libration
42	A_u	Y	0.38	N	0.00	PtI_6 translation
50	A_u	Y	16.37	N	0.00	ν_6
55	A_u	Y	0.12	N	0.00	ν_6
55	B_u	Y	5.21	N	0.00	ν_6
58	B_u	Y	5.23	N	0.00	ν_6
59	A_g	N	0.00	Y	109.63	K translation
61	A_u	Y	3.04	N	0.00	ν_6
62	A_g	N	0.00	Y	200.84	ν_5
62	B_u	Y	58.11	N	0.00	ν_6
62	B_g	N	0.00	Y	238.77	ν_5
63	B_g	N	0.00	Y	19.77	K translation
66	A_g	N	0.00	Y	156.26	ν_5
69	B_u	Y	73.11	N	0.00	K translation
71	A_u	Y	72.26	N	0.00	K translation
73	B_u	Y	141.67	N	0.00	K translation
75	B_g	N	0.00	Y	3.64	ν_5
79	B_g	N	0.00	Y	118.51	ν_5
82	A_g	N	0.00	Y	231.06	ν_5
82	A_u	Y	2.20	N	0.00	ν_4
85	A_u	Y	25.95	N	0.00	ν_4
86	B_u	Y	0.50	N	0.00	ν_2
89	A_g	N	0.00	Y	508.20	K translation
90	A_u	Y	0.11	N	0.00	K translation
93	A_u	Y	0.67	N	0.00	ν_4
93	B_u	Y	4.32	N	0.00	ν_4
94	B_g	N	0.00	Y	263.17	K translation
99	B_u	Y	16.32	N	0.00	ν_4
105	A_g	N	0.00	Y	246.08	K translation
109	A_u	Y	55.18	N	0.00	K translation

109	A_u	Y	55.18	N	0.00	K translation
112	B_g	N	0.00	Y	30.10	K translation
125	A_g	N	0.00	Y	2331.98	ν_2
125	B_g	N	0.00	Y	1542.79	ν_2
126	B_g	N	0.00	Y	2475.74	ν_2
129	B_g	N	0.00	Y	337.51	ν_2
145	B_g	N	0.00	Y	25.89	ν_1
148	A_g	N	0.00	Y	10508.35	ν_1
176	A_u	Y	3.19	N	0.00	ν_3
176	B_u	Y	0.10	N	0.00	ν_3
176	A_u	Y	0.50	N	0.00	ν_3
178	B_u	Y	23.54	N	0.00	ν_3
178	A_u	Y	23.32	N	0.00	ν_3
180	B_u	Y	23.85	N	0.00	ν_3

Table S9 Correlation table for $K_2[PtI_6]$ in *Pmnc*.

Ion	*n*	Free ion		Crystal	Translations		Librations		Intramolecular		Factor group D_{4h}	Total[1]
		Sym.[2]	Rep.	Site[3]	Rep.	No.	Rep.	No.	Rep.	No.		
K	4			D_2	$(B_1 + B_2 + B_3)$	4					$(A_{2g} + B_{2g} + A_{2u} + B_{2u} + E_g + E_u)$	$A_{2g} + B_{2g} + A_{2u} + B_{2u} + 2E_g + 2E_u$
PtI_6	2	O_h	T_{1u}	C_{4h}	$(A_u + E_u)$	2					$(A_{1u} + A_{2u} + E_u)$	$A_{1u} + A_{2u} + 2E_u$
	2	O_h	T_{1g}	C_{4h}	$(A_g + E_g)$			2			$(A_{1g} + A_{2g} + E_g)$	$A_{1g} + A_{2g} + 2E_g$
	2	O_h	A_{1g} (ν_1)	C_{4h}					A_g	2	$(A_{1g} + A_{2g})$	$A_{1g} + A_{2g}$
	2	O_h	E_g (ν_2)	C_{4h}					$(A_g + B_g)$	2	$(A_{1g} + A_{2g} + B_{1g} + B_{2g})$	$A_{1g} + A_{2g} + B_{1g} + B_{2g}$
	2	O_h	T_{1u} (ν_3)	C_{4h}					$(A_u + E_u)$	2	$(A_{1u} + A_{2u} + E_u)$	$A_{1u} + A_{2u} + 2E_u$
	2	O_h	T_{1u} (ν_4)	C_{4h}					$(A_u + E_u)$	2	$(A_{1u} + A_{2u} + E_u)$	$A_{1u} + A_{2u} + 2E_u$
	2	O_h	T_{2g} (ν_5)	C_{4h}					$(B_g + E_g)$	2	$(B_{1g} + B_{2g} + E_g)$	$B_{1g} + B_{2g} + 2E_g$
	2	O_h	T_{2u} (ν_6)	C_{4h}					$(B_u + E_u)$	2	$(B_{1u} + B_{2u} + E_u)$	$B_{1u} + B_{2u} + 2E_u$

[1] Total is the product of the column "No." and the factor group.
[2] Sym. = symmetry, Rep. = irreducible representation of the point group, No. = number.
[3] Symmetry of the site occupied by the ion in the crystal.

Total = $3A_{1g} + 3A_{1u} + 4A_{2g} + 4A_{2u} + 2B_{1g} + 1B_{1u} + 3B_{2g} + 2B_{2u} + 6E_g + 10E_u$

A_{2u} and E_u are infrared allowed; A_{1g}, B_{1g}, B_{2g} and E_g are Raman allowed; A_{1u}, A_{2g}, B_{1u} and B_{2u} are forbidden in both the infrared and Raman spectra.

Table S10 Correlation table for $K_2[PtI_6]$ in $P2_1/c$.

Ion	*n*	Free ion		Crystal							Factor group	Total[1]
					Translations		Librations		Intramolecular		C_{2h}	
		Sym.[2]	Rep.	Site[3]	Rep.	No.	Rep.	No.	Rep.	No.		
K	4			C_1	A	3					$(A_g + B_g + A_u + B_u)$	$3A_g + 3B_g + 3A_u + 3B_u$
PtI_6	2	O_h	T_{1u}	C_i	A_u	3					$(A_u + B_u)$	$3A_u + 3B_u$
	2	O_h	T_{1g}	C_i			A_g	3			$(A_g + B_g)$	$3A_g + 3B_g$
	2	O_h	A_{1g} (ν_1)	C_i					A_g	1	$(A_g + B_g)$	$A_g + B_g$
	2	O_h	E_g (ν_2)	C_i					A_g	2	$(A_g + B_g)$	$2A_g + 2B_g$
	2	O_h	T_{1u} (ν_3)	C_i					A_u	3	$(A_u + B_u)$	$3A_u + 3B_u$
	2	O_h	T_{1u} (ν_4)	C_i					A_u	3	$(A_u + B_u)$	$3A_u + 3B_u$
	2	O_h	T_{2g} (ν_5)	C_i					A_g	3	$(A_g + B_g)$	$3A_g + 3B_g$
	2	O_h	T_{2u} (ν_6)	C_i					A_u	3	$(A_u + B_u)$	$3A_u + 3B_u$

[1] Total is the product of the column "No." and the factor group.
[2] Sym. = symmetry, Rep. = irreducible representation of the point group, No. = number.
[3] Symmetry of the site occupied by the ion in the crystal.

Total = $12A_g + 15A_u + 12B_g + 15B_u$

A_u and B_u are infrared allowed; A_g and B_g are Raman allowed.

Table S11 Experimental transition energies of ν_5 and ν_6 for MF_6 molecules.

Compound	ν_5 / cm^{-1}	ν_6 / cm^{-1}	Method	Ref
SF_6	525	347	Gas phase, Raman, overtone/combination	[14]
	523	346	Gas phase, Raman, overtone/combination	[15]
	524	348	Gas phase, infrared, Raman, overtone/combination	[16]
	524	351	Gas phase, infrared, fundamental, combination	[30]
SeF_6	405	264	Gas phase, Raman, overtone/combination	[14]
	402	263	Gas phase, Raman, overtone/combination	[15]
TeF_6	314	197	Gas phase, Raman, overtone/combination	[14]
	312	201	Gas phase, Raman, overtone/combination	[15]
MoF_6	318	116	Gas phase, Raman, overtone/combination	[14]
	318	114	Gas phase, Raman, overtone/combination	[15]
	317	117	Gas phase, Raman, overtone/combination	[19]
	320	140	Solid state, Raman, fundamental, overtone	[29]
	322^{a}	234	Solid state, infrared, overtone/combination	[28]
WF_6	320	127	Gas phase, Raman, overtone/combination	[14]
	320	126	Gas phase, Raman, overtone/combination	[15]
	320	127	Gas phase infrared, Raman, overtone/combination	[20]
	323	147	Solid state, Raman, fundamental, overtone	[29]
TcF_6	297	145	Gas phase, Raman, overtone/combination	[14]
ReF_6	295	147	Gas phase, Raman, overtone/combination	[14]
	296	167	Gas phase and solid state, NIR vibronic structure	[15]
	296	165	Gas phase, NIR vibronic structure	[27]
RuF_6	283	186	Gas phase, Raman, overtone/combination	[13]
OsF_6	276	205	Gas phase, Raman, overtone/combination	[13]
RhF_6	269	192	Gas phase, Raman, overtone/combination	[14]
IrF_6	267	206	Gas phase, Raman, overtone/combination	[13]
PtF_6	242	211	Raman, overtone/combination	[14]
UF_6	202	142	Raman, overtone/combination	[14]
	200	141	Gas phase infrared, Raman, overtone/combination	[17]
	200	144	Matrix isolated, fluorescence	[18]
NpF_6	208	164	Gas phase, Raman, overtone/combination	[14]
	208	169	Matrix isolated, fluorescence	[18]
PuF_6	211	173	Gas phase, Raman, overtone/combination	[13]
	209	177	Matrix isolated, fluorescence	[18]
$Cs_2[TiCl_6]$	186	139	Solid state, infrared, overtone/combination	[21]
$Cs_2[ZrCl_6]$	161	112	Solid state, infrared, overtone/combination	[21]
$Cs_2[HfCl_6]$	167	110	Solid state, infrared, overtone/combination	[21]
$Cs[NbCl_6]$	179^{a}	118	Solid state, infrared, overtone/combination	[21]
$Cs[TaCl_6]$	184^{a}	101	Solid state, infrared, overtone/combination	[21]
$Cs[TaBr_6]$	116^{a}	75	Solid state, infrared, overtone/combination	[21]
$[AsF_6][ClF_6]^{b}$	517	347	Solid state, Raman, overtone/combination	[22]
$K[AsF_6]$	376	232	Solid state, infrared, fundamental	[23]

$K[SbF_6]$	288[a]	179[a]	Solid state, infrared, fundamental	[24]
WCl_6	182	97	CS_2 solution, infrared, overtone/combination	[25]

[a]Average of factor group components. [b]Values are for the $[ClF_6]^+$ ion.

Table S12 Plane wave cut-offs (eV) and Monkhorst-Pack (MP) grids used in the calculations of the $A_x[MHal_y]$ complexes.

	$K_2[SiF_6]$	$Na_2[SiF_6]$	$K_2[TiF_6]$	$K[PF_6]$	$Na_3[AlF_6]$	$K_2[ReCl_6]$	$K_2[PtCl_6]$	$K_2[PtI_6]$	$K_2[PtI_6]$
SG	$Fm\bar{3}m$	$P321$	$P\bar{3}m1$	$C2/n$	$P2_1/n$	$Fm\bar{3}m$	$Fm\bar{3}m$	$Pmnc$	$P2_1/c$
Cut-off	840	940	940	940	940	830	480	830	1000
MP grid	8 × 8 × 8	4 × 4 × 8	4 × 4 × 5	4 × 4 × 4	5 × 5 × 4	8 × 8 × 8	8 × 8 × 8	8 × 8 × 5	8 × 8 × 5
No. *k*-points	60	20	14	20	20	60	60	30	80
No. imaginary modes	0	0	0	0	0	3	0	2	0

References

[1] J. K. Cockcroft, Neutron-scattering studies of order-disorder transitions in hexafluoride salts ABF_6, Thesis (D.Phil.), University of Oxford, 1985.

[2] S. F. Parker, A. J. Ramirez-Cuesta, L. Daemen, The structure and vibrational spectroscopy of cryolite, Na_3AlF_6. *RSC Advances* 2020, **10**, 25856–25863.

[3] https://icsd.products.fiz-karlsruhe.de/sites/default/files/ICSD/documents/brochures/A-Focus-on-Crystallography.pdf

[4] Schefer, J.; Schwarzenbach, D.; Fischer, P.; Koetzle, Th.; Larsen, E K.; Haussühl, S.; Rüdlinger, M.; Mcintyre, G.; Birkedal, H.; Bürgi, H.-B. Neutron and X-ray diffraction study of the thermal motion in K_2PtCl_6 as a function of temperature. Acta Cryst. **1998**, B54, 121-128.

[5] Adams, D. M.; Gebbie, H. A. Absorption spectra of some inorganic complex halides by far infra-red interferometry. *Spectrochim. Acta* **1963**, *19*, 925-930.

[6] Hiraishi, J.; Shimanouchi, T. Lattice vibrations and the force field of K_2PtCl_4, K_2PdCl_4 and K_2PtCl_6. *Spectrochim. Acta* **1966**, *22*, 1483-1491.

[7] Debeau, M.; Krauzman, M. Spectres de vibration de complexes métalliques hexahalogénés cristallisés. *Comptes Rendu Seances Acad. Sci., Ser. B*, **1967**, *264*, 1724-1727.

[8] Hendra, P. J.; Park, P. J. D. The Raman and far infra-red spectra of some octahedral transition metal anions in the crystalline phase. *Spectrochim. Acta A* **1967**, *23*, 1635-1640.

[9] Adams, D. M.; Morris, D. M. Vibrational spectra of halides and complex halides. Part I. Hexahalogenoplatinates. *J. Chem. Soc. A* **1967**, 1666-1668.

[10] Bosworth, Y. M.; Clark, R. J. H. Intensity studies on the Raman-active fundamentals of hexahalogenoanions of second- and third-row transition and non-transition metals. The calculation of parallel and perpendicular bond polarisability derivatives *J. Chem. Soc., Dalton Trans*. **1974**, 1749-1761.

[11] Parker, S. F.; Forsyth, J. B. K_2MCl_6: (M = Pt, Ir) Location of the silent modes and forcefields. *J. Chem. Soc. Faraday Trans.* **1998**, *94*, 1111-1114.

[12] Hamaguchi, H.; Harada, I.; Shimanouchi, T. Resonance Raman effect of octahedral complex ions, $PtBr_6^{2-}$ and PtI_6^{2-}. *J. Raman Spec*. **1974**, *2*, 517-528.

[13] B. Weinstock and G. Goodman, Vibrational properties of hexafluoride molecules, *Advan. Chem. Phys*. 1965, **9**, 169-319.

[14] H. H. Claassen, G. L. Goodman, J. H. Holloway and H. Selig, Raman spectra of MoF_6, TcF_6, ReF_6, UF_6, SF_6, SeF_6, and TeF_6 in the vapor state, *J. Chem. Phys.* 1970, **53**, 341-348.

[15] Y. M. Bosworth, R. J. H. Clark and D. M. Rippon, The vapor phase Raman spectra, Raman band contour analyses, and Coriolis constants of the spherical top molecules MF_6 (M = S, Se, Te, Mo, W, or U), $M(CH_3)_4$, (M = C, Si, Ge, Sn, or Pb), P_4, As_4 and OsO_4, *J. Mol. Spec.* 1973, **46**, 240-255.

[16] C. Chapados and G. Birnbaum, Infrared absorption of SF_6 from 32 to 3000 cm^{-1} in the gaseous and liquid states, *J. Mol. Spec.* 1988, **132**, 323-351.

[17] R. S. McDowell, L. B. Asprey and R. T. Paine, Vibrational spectrum and force field of uranium hexafluoride, *J. Chem. Phys.* 1974, **61**, 3571-3580.

[18] R. N. Mulford, H. J. Dewey and J. E. Barefield II, Fluorescence and absorption spectroscopy of the near-infrared vibronic transitions in matrix-isolated NpF_6, *J. Chem. Phys.* 1991, **94**, 4790-4796.

[19] R. S. McDowell, R. J. Sherman, L. B. Asprey and R. C. Kennedy, Vibrational spectrum and force field of molybdenum hexafluoride, *J. Chem. Phys.* 1975, **62**, 3974-3978.

[20] R. S. McDowell and L. B. Asprey, Coriolis constants of spherical-top molecules from low-temperature infrared studies of vapor band contours. Application to the force field of tungsten hexafluoride, *J. Mol. Spec.* 1973, **48**, 254-265.

[21] W. van Bronswyk, R. J. H. Clark and L. Maresca, Infrared spectra, laser Raman spectra, and force constants of the metal-hexahalo species $R_2M^{IV}X_6$, $RM^{V}X_6$ [R = $(C_2H_5)_4N$ or Cs; M^{IV} = Ti, Zr, or Hf; M^{V} = Nb or Ta; X = Cl or Br], and WCl_6, *Inorg. Chem.* 1969, **8**, 1395-1401.

[22] K. O. Christe, W. W. Wilson and E. C. Curtis, Coordinatively saturated complex fluoro cations. Synthesis and characterization of $C1F_6^+AsF_6^-$ and $ClF_6^+SbF_6^-$, *Inorg. Chem.* 1983, **22**, 3056-3060.

[23] A. M. Heyns and C. W. T. Pistorius, Vibrational spectra, high-pressure polymorphism and force constants of $KAsF_6$, *Spectrochim. Acta A* 1975, **31**, 1293-1301.

[24] A. M. Heyns and C. W. T. Pistorius, Polymorphism, high-pressure phase diagram and vibrational spectra of $KSbF_6$, *Spectrochim. Acta A* 1976, **32**, 535-545.

[25] J. C. Evans and G. Y-S. Lo, A vibrational assignment for tungsten hexachloride, *J. Mol. Spec.* 1968, **26**, 147-149.

[26] J. C. D. Brand, G. L. Goodman and B. Weinstock, The near-infrared band system of rhenium hexafluoride, *J. Mol. Spec.* 1971, **38**, 449-463.

[27] R. McDiarmid, Jahn-Teller effects in the $^2E_{52g}$⟵$^2G_{32g}$ transition of rhenium hexafluoride, *J. Mol. Spec.* 1971, **38**, 495-502.

[28] K. H. Hellberg, A. Müller and O. Glemser, Das Infrarotspektrum von festem CrF_6, MoF_6 und OsF_6 bei tiefen Temperaturen, *Z. Naturforschg*. 1966, **21b**, 118-121.

[29] E. R. Bernstein and G. R. Meredith, Vibrational spectra of transition metal hexafluoride crystals. III. Exciton band structures of MoF_6, WF_6 and UF_6, *Chem. Phys.* 1977, **24**, 311-325.

[30] C. Chapados and G. Birnbaum, The forbidden far-infrared ν_6 band of SF_6, *J. Mol. Spec.* 1984, **105**, 206-214.
